# A novel recombination protein C12ORF40/REDIC1 is required for meiotic crossover formation

**DOI:** 10.1038/s41421-023-00577-5

**Published:** 2023-08-23

**Authors:** Suixing Fan, Yuewen Wang, Hanwei Jiang, Xiaohua Jiang, Jianteng Zhou, Yuying Jiao, Jingwei Ye, Zishuo Xu, Yue Wang, Xuefeng Xie, Huan Zhang, Yang Li, Wei Liu, Xiangjun Zhang, Hui Ma, Baolu Shi, Yuanwei Zhang, Muhammad Zubair, Wasim Shah, Zhipeng Xu, Bo Xu, Qinghua Shi

**Affiliations:** 1 Division of Reproduction and Genetics, First Affiliated Hospital of USTC, Hefei National Research Center for Physical Sciences at the Microscale, the CAS Key Laboratory of Innate Immunity and Chronic Disease, School of Basic Medical Sciences, Division of Life Sciences and Medicine, Biomedical Sciences and Health Laboratory of Anhui Province, Institute of Health and Medicine, Hefei Comprehensive National Science Center, University of Science and Technology of China, Hefei, Anhui China; 2 Institute of Andrology, Nanjing Drum Tower Hospital, The Affiliated Hospital of Nanjing University Medical School, Nanjing, Jiangsu China

**Keywords:** Meiosis, Homologous recombination

## Abstract

During meiosis, at least one crossover must occur per homologous chromosome pair to ensure normal progression of meiotic division and accurate chromosome segregation. However, the mechanism of crossover formation is not fully understood. Here, we report a novel recombination protein, C12ORF40/REDIC1, essential for meiotic crossover formation in mammals. A homozygous frameshift mutation in *C12orf40* (c.232_233insTT, p.Met78Ilefs*2) was identified in two infertile men with meiotic arrest. Spread mouse spermatocyte fluorescence immunostaining showed that REDIC1 forms discrete foci between the paired regions of homologous chromosomes depending on strand invasion and colocalizes with MSH4 and later with MLH1 at the crossover sites. *Redic1* knock-in (KI) mice homozygous for mutation c.232_233insTT are infertile in both sexes due to insufficient crossovers and consequent meiotic arrest, which is also observed in our patients. The foci of MSH4 and TEX11, markers of recombination intermediates, are significantly reduced numerically in the spermatocytes of *Redic1* KI mice. More importantly, our biochemical results show that the N-terminus of REDIC1 binds branched DNAs present in recombination intermediates, while the identified mutation impairs this interaction. Thus, our findings reveal a crucial role for C12ORF40/REDIC1 in meiotic crossover formation by stabilizing the recombination intermediates, providing prospective molecular targets for the clinical diagnosis and therapy of infertility.

## Introduction

In mammals, gametes are produced through a specialized cell division called meiosis that halves the genome of the germ cell. During meiosis, each pair of homologous chromosomes must obtain at least one crossover to ensure their correct congression to the metaphase I equatorial plate, progression of meiotic division, and subsequent accurate chromosome segregation^1,2^. Reduction of crossover formation causes not only meiotic arrest in mice but also gametogenesis failure and infertility in humans^3–7^, a complex disease that affects ~8%–12% of reproductive-age couples worldwide^8^. Even when the reduction in recombination occurs only between sex chromosomes in males, apoptosis of spermatocytes and a decreased sperm count will occur, which ultimately affects fertility^9^. Thus, proper crossover formation is crucial for gamete production. However, the regulatory mechanism of crossover formation is not fully understood.

Synapsis and recombination are two interdependent events that occur during meiotic prophase I. The synaptonemal complex (SC) is a proteinaceous zipper-like structure that holds homologous chromosomes together to facilitate meiotic recombination and crossover formation^10^. Meiotic recombination begins with the generation of programmed DNA double-strand breaks (DSBs) catalyzed by the topoisomerase VI complex proteins SPO11 and TOP6BL^11,12^. These DSBs are then resected to generate 3' single-strand DNA (ssDNA) overhangs coated by the RPA complex. Then, RAD51 and DMC1 recombinases replace RPA to catalyze the homology search and strand invasion into the homologous chromosome, leading to the formation of displacement loops (D-loops). These recombination intermediates are then stabilized to promote the pairing and synapsis of homologous chromosomes. Subsequently, most DSBs are repaired as noncrossover products via the synthesis-dependent strand annealing (SDSA) pathway. Only a small fraction of recombination intermediates are transformed into double Holliday junctions (dHJs) and resolved mostly to crossovers^1,13^. To date, two types of crossovers have been described. One depends on the MutL homologs MLH1 and MLH3 and is interference-sensitive, called class I crossovers, contributing to most of the total crossovers in mammalian germ cells^14–16^. The other, called class II crossovers, depends on MUS81-EME1 and exhibits no interference^17–19^.

In mice, ~250 DSBs are produced per spermatocyte, with 90% of them repaired as noncrossover and only 10% repaired as crossover (~24 crossovers per cell), suggesting that the DSB repair pathways are strictly regulated^20–23^. The key to this regulation is based on the stability of recombination intermediates. ZMM proteins, a group of functionally related and evolutionarily conserved proteins, are believed to bind recombination intermediates and promote meiotic crossover formation^24^. To date, eight ZMM proteins (Zip1, Zip2, Zip3, Zip4, Spo16, Msh4, Msh5, and Mer3) have been identified in budding yeast^25^, and their orthologs have also been clarified in mammals^26^. Except for SYCP1 (ortholog of Zip1) as the transverse filament of SC, other ZMM proteins are crucial for the development of single-end invasions (SEIs) to dHJs, thus promoting meiotic recombination. After strand invasion, SHOC1 (ortholog of Zip2) is recruited to nascent D-loops and promotes DNA synthesis; it also recruits SPO16, which is required for the stabilization of SHOC1 and proper localization of TEX11 (ortholog of Zip4) and MSH4^26,27^. MSH4 and MSH5 form a clamp-like heterodimer to bind Holliday junctions and maintain a stable connection between homologous chromosomes, which is essential for synapsis initiation in the zygotene stage^28,29^. MSH4-MSH5 heterodimers are also responsible for the recruitment of MLH1 and MLH3 to the crossover sites in pachytene^30^. TEX11 is an X-linked factor that promotes initiation and/or maintenance of chromosome synapsis and crossover formation^5,31^. Similarly, depletion of HFM1 (ortholog of Mer3), a 3′–5′ helicase, in mouse spermatocytes, resulted in incomplete synapsis and drastic loss of crossovers^6^. Following synapsis, the stability of recombination intermediates is further regulated by RNF212-mediated SUMOylation and HEI10-mediated ubiquitylation^32–35^. Finally, those recombination intermediates bound by RNF212 and HEI10 (ortholog of Zip3) further recruit CDK2 and MLH1-MLH3, and are eventually repaired into crossover products.

Here, we demonstrate that a previously uncharacterized protein, C12ORF40, promotes meiotic recombination and crossover formation by stabilizing recombination intermediates. A frameshift mutation c.232_233insTT in this gene was identified via whole-exome sequencing (WES) in two unrelated azoospermic men with meiotic arrest and is conserved in humans and mice. CN725425, the murine ortholog of human C12ORF40, is highly expressed in the testis and fetal ovary, and localizes to the paired regions of homologous chromosomes as discrete foci in a DSB-dependent manner. The knock-in (KI) mice mimicking the patients’ mutation showed mild synaptic defects but a drastic reduction in class I crossovers, and consequently metaphase I arrest. We thus named this gene “*Redic1*”. In the *Redic1* mutant mice, pachytene spermatocytes showed a great decrease in recombination intermediates. Thus, our study demonstrates that C12ORF40/REDIC1 is essential for meiotic progression and crossover formation by stabilizing recombination intermediates in mammals.

## Results

### Identification of *C12orf40* mutation by WES from two nonobstructive azoospermic patients with meiotic arrest

**Fig. 1:**
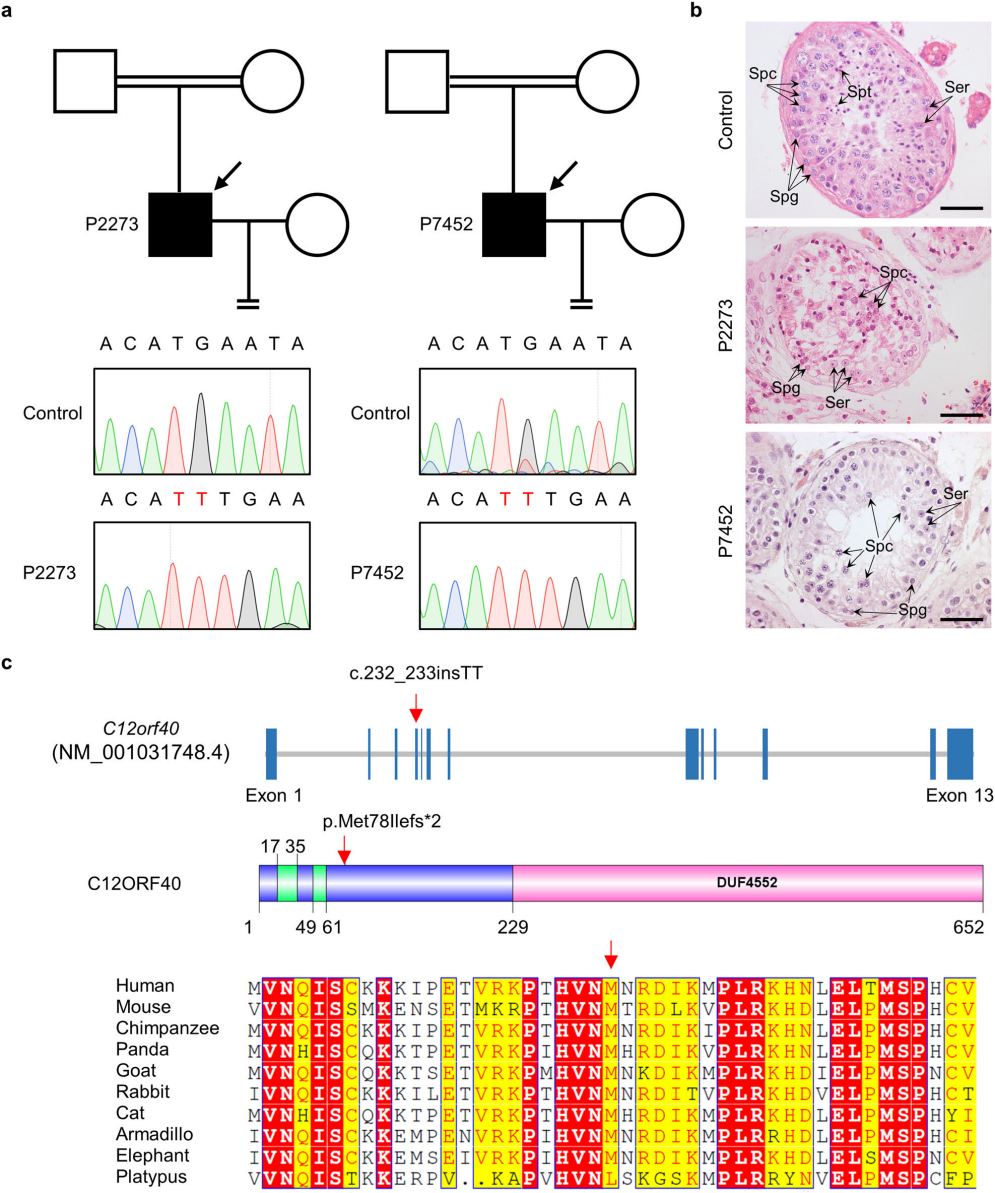
Identification of a homozygous frameshift mutation in *C12orf40* from two NOA-affected men born to consanguineous parents. **a** Pedigrees of two Chinese families with a *C12orf40* mutation. The double horizontal lines indicate the consanguineous marriage. Squares and circles denote male and female members, respectively. Solid symbols indicate the members with nonobstructive azoospermia. Members indicated by arrows were selected for WES. Sanger sequencing chromatograms of *C12orf40* are shown at the bottom. The 2-bp insertion in *C12orf40* is marked in red. **b** Histological analysis of human testicular sections by hematoxylin and eosin staining. A man who was diagnosed with obstructive azoospermia served as the control. Spg, spermatogonium; Spc, spermatocyte; Spt, spermatid; Ser, Sertoli cell. Scale bars: 50 μm. **c** Location and conservation of the C12ORF40 mutation. The gene composition is based on the Ensembl database (GRCh38, transcript ID: ENST00000324616.9; NCBI RefSeq ID: NM_001031748.4). The blue solid squares represent exons. The domains were predicted by the SMART web server. The conservation of the mutated amino acid is evaluated by the sequence alignment of orthologs from the indicated species. The red arrow shows the identified mutation. The results of the full-length protein multiple sequence alignment are shown in Supplementary Fig. S2.

Two Chinese men with nonobstructive azoospermia (P2273 and P7452), aged 40 and 30, respectively, are from two unrelated consanguineous families (Fig. 1a). They showed normal height and body weight, with an unremarkable medical history except for suffering from primary infertility. They have normal karyotypes (46, XY) with no Y-chromosome microdeletions. Semen analysis showed no sperm present in the semen of individual P2273, and his reproductive hormones were within the normal range (Supplementary Table S1). Their testicular biopsies were obtained and examined (Fig. 1b). For the control, spermatogenic cells, including spermatogonia, spermatocytes, and spermatozoa, are evident in the seminiferous tubules, but for individuals P2273 and P7452, all the analyzed seminiferous tubules contain spermatogonia and spermatocytes but lack spermatids and spermatozoa. These observations confirmed that these two men are nonobstructive azoospermic due to meiotic arrest.

To explore the genetic causes of their infertility, we performed WES of the genomic DNA of these two affected men. Through a series of filtrations and bioinformatics analyses, we initially obtained only one frameshift mutation in *C12orf40* (c.232_233insTT, p.Met78Ilefs*2) in individual P7452 and eight mutations in seven genes in individual P2273, including the same *C12orf40* mutation as in P7452 (Supplementary Fig. S1a). The mutations in P2273 included two frameshift mutations and six missense mutations. Given that the frameshift mutations in *C12orf40* (c.232_233insTT, p.Met78Ilefs*2) and *PP2D1* (c.719delT, p.Met240fs) are expected to result in deletion of the vast majority of regions of both proteins, they were prioritized. Furthermore, considering that both affected men exhibited a spermatocyte developmental arrest phenotype, we analyzed the expression patterns of their murine orthologs using testis single-cell transcriptome data^36^ and found that *CN725425*/*Redic1* (the murine ortholog of *C12orf40*) was highly expressed in spermatocytes of early meiotic prophase I, whereas *Pp2d1* was dominantly expressed in round spermatids and mice homozygous for a null allele, *Pp2d1*^em1Zuk^, exhibit normal male fertility (MGI: 6693655)^37,38^. Thus, we think that the homozygous frameshift mutation in *C12orf40* is the candidate disease-causing mutation for infertility in these affected men.

The 2-bp insertion in *C12orf40* was subsequently validated by Sanger sequencing and is predicted to result in a truncated protein with 78 amino acids (the full-length C12ORF40 protein contains 652 amino acids) that would lose its C-terminal domain (Fig. 1a, c). Moreover, this mutation is absent from the 1000 Genome Project database and has a low allele frequency in the gnomAD database (4.911 × 10^−5^). Furthermore, this mutation was located within the homozygosity region in the genome of both affected men (Supplementary Fig. S1b), corroborating that they were offspring of consanguineous parents.

### REDIC1 is dominantly expressed in gonads and located in the paired regions of homologous chromosomes

*C12orf40* is a novel gene that encodes an uncharacterized protein that conserves in mammals, including humans, chimpanzees, and mice (Fig. 1c; Supplementary Fig. S2). The RNA-sequencing results from the NCBI database showed that *C12orf40* is highly expressed in human testicular tissue^39,40^. To confirm this and better understand the role of *C12orf40*, we first examined the expression of *Redic1* in different mouse tissues by real-time quantitative polymerase chain reaction (RT-qPCR). High levels of *Redic1* cDNA can be detected in adult testes of male mice and embryonic ovaries from 14.5 days post-coitum (dpc) female mice, with no expression or low levels in other tissues (Supplementary Fig. S3), suggesting its potential role in meiosis.

**Fig. 2:**
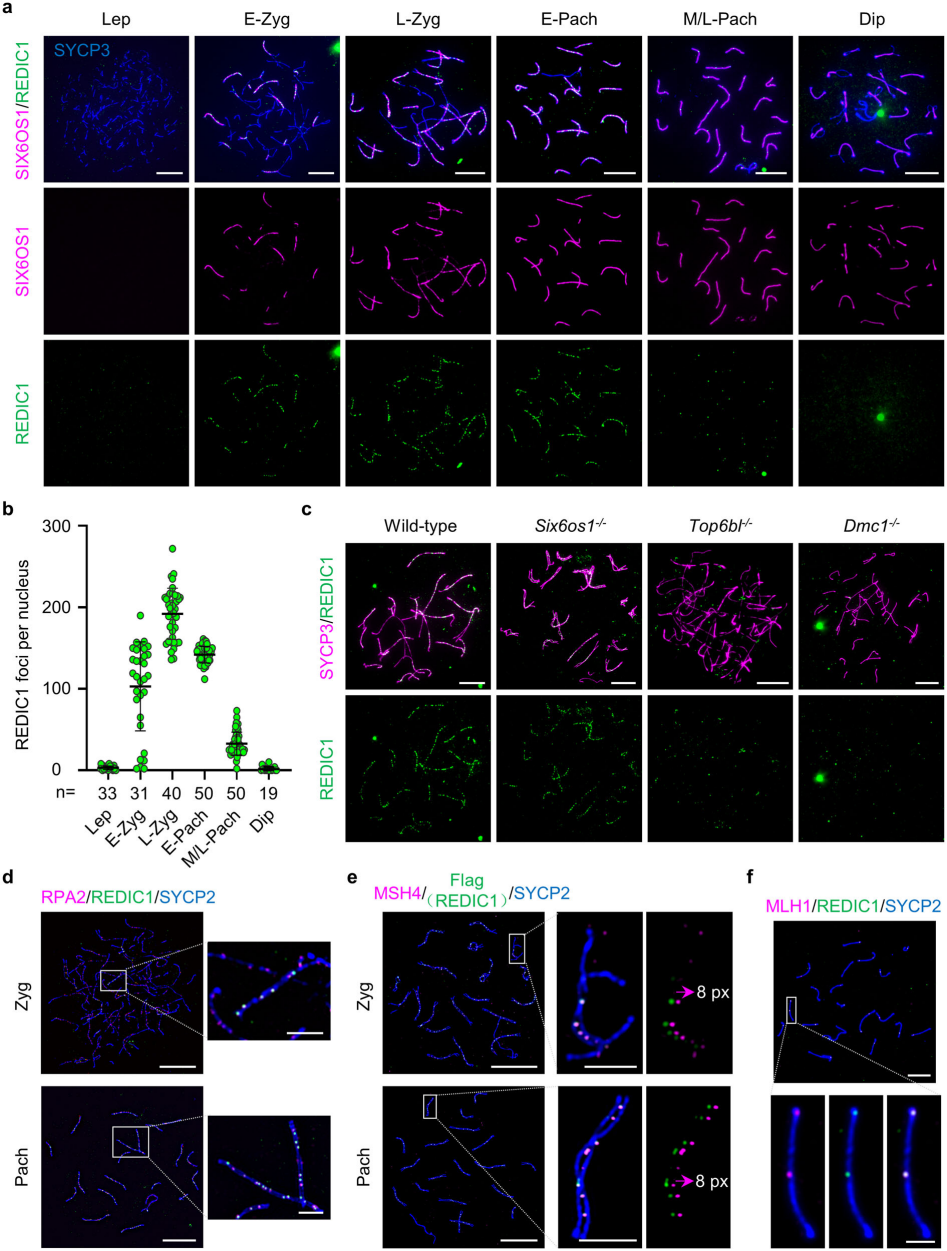
REDIC1 is localized to recombination intermediates in meiotic prophase cells. **a** Representative spread spermatocytes of 30 dpp WT mice stained with antibodies against SYCP3 (blue), REDIC1 (green), and SIX6OS1 (magenta). Lep, leptotene; E-Zyg, early zygotene; L-Zyg, late zygotene; E-Pach, early pachytene; M/L-Pach, mid- or late pachytene; Dip, diplotene. Scale bars: 10 μm. **b** Quantification of the number of REDIC1 foci on chromosome axes at the indicated substages from WT mice. *n*, the total number of cells analyzed from two animals. The bars represent mean ± SD. **c** Representative images of zygotene (like) spermatocyte spreads from adult WT, *Six6os1*^–/–^, *Top6bl*^–/–^, and *Dmc1*^–/–^ mice stained for SYCP3 (magenta) and REDIC1 (green). The experiments were repeated two times with similar results. Scale bars: 10 μm. **d** Representative images of surface-spread chromosomes from zygotene and pachytene mouse spermatocytes stained for RPA2 (magenta), REDIC1 (green), and the chromosomal axis marker SYCP2 (blue). The areas in the white rectangles are enlarged. **e** Structured illumination microscopy of spread zygotene and pachytene spermatocytes of *Redic1*-Flag/Myc mouse stained for MSH4 (magenta), Flag (green), and chromosomal axis marker SYCP2 (blue). The areas in the white rectangles are enlarged. The panel on the far right shows the magenta and green signals shifted by 8 pixels. Images are representatives from experiments with two adult animals. **f** Representative images of surface-spread chromosomes from a mid-pachytene spermatocyte stained for MLH1 (magenta), REDIC1 (green), and SYCP2 (blue) in WT mice. The magnified panels show a pair of synapsed chromosomes on which two MLH1 foci colocalized with two REDIC1 signals. The experiments were performed at least twice with similar results. Scale bars in **d**, **e**, and **f** are 10 μm for the original images and 2 μm for the enlarged images, respectively.

To investigate the localization of REDIC1 during meiosis, we performed immunofluorescence staining on surface-spread mouse spermatocyte nuclei using a customized antibody that specifically recognizes the C-terminus of REDIC1 (Fig. 2a; Supplementary Fig. S4). For staging, the spermatocyte nuclei were costained with antibodies against SC lateral element protein SYCP3 and central element protein SIX6OS1 simultaneously. Cells with short SYCP3 fragments and no SIX6OS1 signal were identified as leptotene spermatocytes; cells with visible SIX6OS1 threads but incomplete lateral elements (LEs) were clarified as early zygotene spermatocytes; cells with intact LEs that have not yet achieved full synapsis of all autosomes were late zygotene spermatocytes; cells with fully synapsed autosomes and uniform thickness of synapsed axes are clarified as early pachytene spermatocytes; cells with shorter and/or thicker fully synapsed autosomes and short synapsed PAR are identified as mid- or late pachytene spermatocytes; cells with disassembled SIX6OS1 but intact SYCP3 signals with brighter ends are clarified as diplotene spermatocytes. REDIC1 was first detected as foci in the synapsed region of homologous chromosomes from the zygotene stage. The focus number increased with synapsis progression and peaked in late-zygotene spermatocytes (192.1 ± 31.4 per nucleus; *n* = 40). In pachytene spermatocytes, REDIC1 signals, localized along autosomal axes and the synapsed pseudoautosomal region on sex chromosomes, decreased rapidly after early pachytene and were only one or two on each pair of homologs (32.6 ± 14.0 per nucleus; *n* = 50) by mid- or late pachytene, and disappeared in the diplotene stage (Fig. 2b). The localization dynamics of REDIC1 are similar to those of some meiotic DSB repair proteins, such as MSH4^29^. To further characterize the localization of REDIC1, we performed super-resolution imaging using structured illumination microscopy (SIM) and found that REDIC1 foci are located between the paired chromosome axes, mainly on the central elements of the SCs (Supplementary Fig. S5). Similarly, REDIC1 foci can also be detected on the paired regions of homologous chromosomes in zygotene and pachytene oocytes (Supplementary Fig. S6). Collectively, these results suggest a possible role for REDIC1 in meiotic recombination.

### The localization of REDIC1 is dependent on meiotic DSBs and strand invasion

Given the localization pattern of REDIC1 and its relationship with synapsis, we examined the genetic requirements for REDIC1 localization using *Six6os1*^–/–^^41^, *Top6bl*^–/–^^12^, and *Dmc1*^–/–^ mouse models^42,43^. As reported^41^, in *Six6os1*-deficient spermatocytes, homologous chromosomes were able to pair but not synapse because all the known central elements were absent, and the early recombination intermediates marked by MSH4 were formed in *Six6os1*-deficient spermatocytes, although they failed to be processed into late recombination nodules. The *Top6bl*^–/–^ mice did not form meiotic programmed DSBs^12^, and the *Dmc1* knockout mice had almost no strand invasion between homologs^42,43^. Interestingly, in the zygotene spermatocytes of *Six6os1*^–/–^ mice, the number of REDIC1 foci per cell averaged 158.8 ± 43.1 (*n* = 44), which is comparable to that of the wild-type (WT) mice (144.0 ± 41.8; *n* = 21 nuclei), indicating that REDIC1 localization is independent of synapsis. Whereas in the zygotene spermatocytes of *Top6bl*^–/–^ and *Dmc1*^–/–^ mice, the average number of REDIC1 on the axis per cell is much fewer than that of the WT mice (*Top6bl*^–/–^: 9.5 ± 7.6; *n* = 39 nuclei; *Dmc1*^–/–^: 7.9 ± 6.2; *n* = 37 nuclei; Fig. 2c; Supplementary Fig. S7). These findings indicate that REDIC1 chromosomal localization is mainly dependent on meiotic DSBs and interhomolog strand invasion, which is similar to MSH4 but distinct from TEX11 and SPO16, because the latter two proteins are still detected on the chromosome axes of spermatocytes in *Dmc1*^–/–^ mice^26^.

### REDIC1 is first located on recombination intermediates and later colocalizes with MLH1 at the crossover sites

To further clarify the localization of REDIC1, we first performed costaining of REDIC1 with ssDNA-binding proteins, such as RPA2 and DMC1, on surface-spread mouse spermatocytes. RPA2, as a subunit of the RPA complex, has been shown to localize to the ssDNA on resected DSB ends as well as the ssDNA in D-loops formed after strand invasion^44^. Interestingly, in spermatocytes from early zygotene to early pachytene, more than 90% of REDIC1 foci colocalized with RPA2 (Fig. 2d; Supplementary Fig. S8a), while the percentage of RPA2 foci colocalizing with REDIC1 increased from 36.5% to 75.1% (Supplementary Fig. S8b), indicating that REDIC1 localizes on recombination intermediates. In addition, costaining of REDIC1 with DMC1 showed that 20%–30% of REDIC1 foci are colocalized with DMC1 at the zygotene stage, but their colocalization declines quickly after entering the pachytene stage (Supplementary Fig. S9), suggesting that REDIC1 may be recruited before the release of recombinases from recombination intermediates and persist afterward.

Next, we wondered whether REDIC1 colocalizes with ZMM proteins, especially with MSH4, a marker protein for recombination intermediates^28,45^. Due to antibody limitations, we were unable to answer this question in the past. Until recently, we obtained *Redic1*-*Flag/Myc* mice, allowing us to examine it by costaining of Flag with MSH4 and SYCP2 antibodies. By using SIM, we confirmed that both REDIC1 and MSH4 foci localize predominantly between paired homologous chromosome axes from the zygotene onwards (Fig. 2e). In early zygotene spermatocytes, 88.5% of REDIC1 foci colocalized with MSH4, corresponding to 66 REDIC1-MSH4 co-foci per cell. Subsequently, more than 90% of REDIC1 foci colocalized with MSH4 from the late zygotene to the late pachytene stage (Supplementary Fig. S10). To further clarify the relationship between REDIC1 and ZMM proteins, we performed co-immunoprecipitation (co-IP) experiments and found that REDIC1 interacts with MSH5, a partner of MSH4, and with TEX11, but not with HFM1 or RNF212 (Supplementary Fig. S11). These results indicate that REDIC1 localizes on the recombination intermediates, similar to the MutS complex.

More importantly, we also performed costaining of REDIC1 with MLH1, a component of late recombination nodules and widely used class I crossover marker, and found that 81.67% of REDIC1 foci are colocalized with 95.32% of MLH1 foci in mid-pachytene spermatocytes (Fig. 2f; Supplementary Fig. S12). Taken together, we believe that REDIC1 is localized first to recombination intermediates and later colocalizes with MLH1 at crossover sites.

### Male mice homozygous for the *Redic1* KI mutation c.232_233insTT show spermatogenic failure

To explore the functional role of *Redic1* in spermatogenesis and evaluate the pathogenicity of the identified frameshift mutation in the affected men, we generated a *Redic1* KI mouse model that mimicked the identified human *REDIC1* mutation c.232_233insTT using CRISPR/Cas9 technology (Supplementary Fig. S13a). This mutation did not lead to the degradation of mRNA or the production of potentially in-frame reinitiation-translated proteins (Supplementary Fig. S13b, c). Correspondingly, immunofluorescence staining did not show any signals of REDIC1 on chromosome axes of mutant spermatocytes (Supplementary Fig. S13d). Thus, we guess that the mutation may lead to the production of a truncated protein containing only 78 amino acids at the N-terminal end of REDIC1, which cannot be detected using our REDIC1 antibody.

**Fig. 3:**
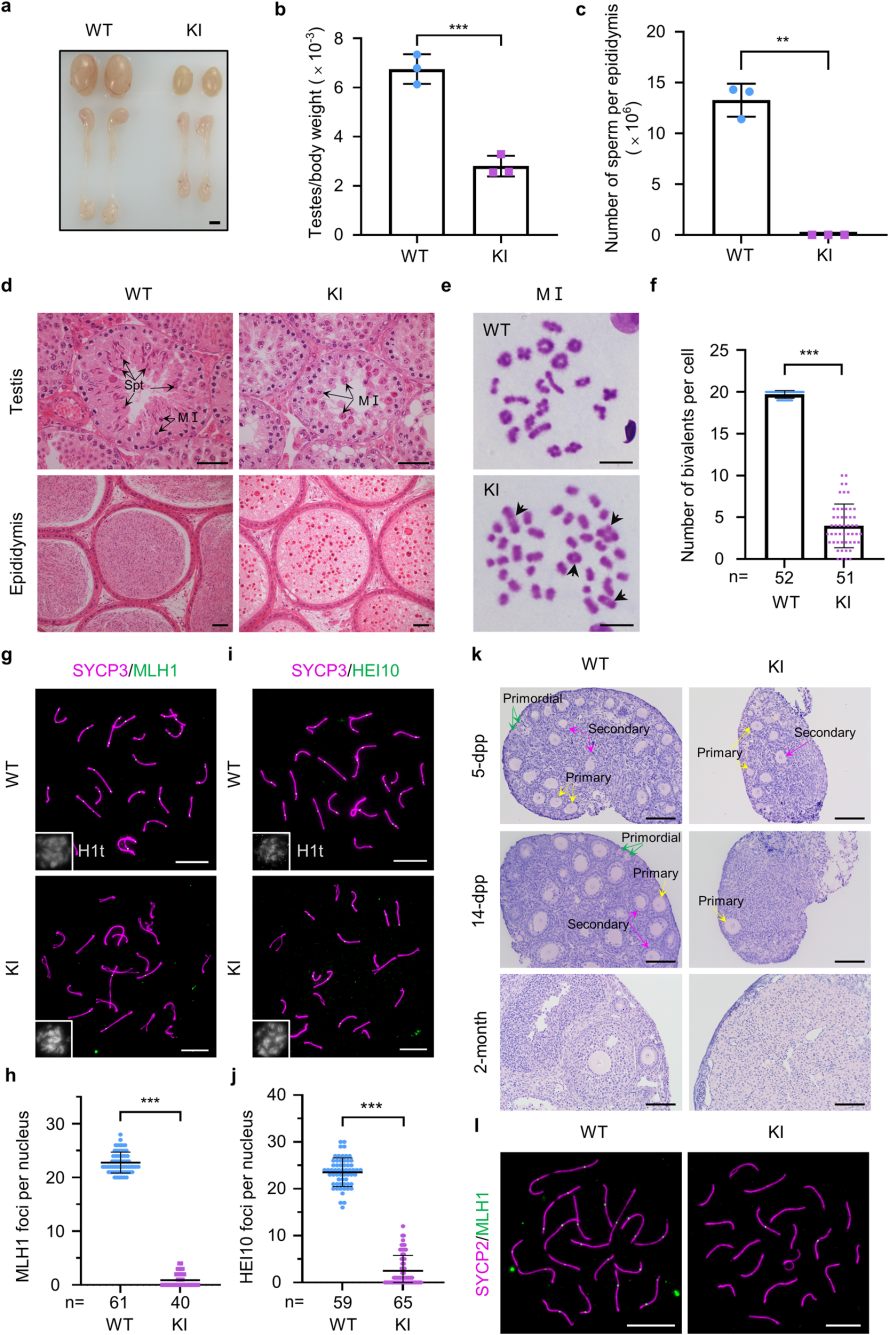
Mouse models mimicking the patients’ mutation exhibited defects in crossover formation. **a** Representative images of testis and epididymis morphology from 2-month-old WT and *Redic1* KI mice. Scale bar: 2 mm. **b**, **c** Ratios of the testis to body weight (**b**) and sperm counts per epididymis (**c**) in 2-month-old WT and *Redic1* KI mice. The data are from three adult mice for each genotype and represent the mean ± SD. **P < 0.01; ***P < 0.001; two-tailed Student’s *t*-test. **d** Representative images of hematoxylin and eosin-stained testicular and epididymal sections from adult *Redic1* KI and WT mice. The metaphase I spermatocytes (MI) and spermatids (spt) are indicated by the arrows. Scale bars: 50 μm. **e** Representative metaphase I (MI) spermatocytes from adult WT and *Redic1* KI mice stained with Giemsa. The bivalent chromosomes in *Redic1* KI mice are indicated by the arrows. Scale bars: 10 μm. **f** Quantification of bivalents per metaphase I spermatocyte from 2-month-old WT and KI mice. *n*, the total number of MI cells scored from two animals for each genotype. Data are presented as the mean ± SD. ***P < 0.001; Welch’s *t*-test. **g** Representative spreads of mid-pachytene spermatocytes from WT and KI mice stained for SYCP3 (magenta), MLH1 (green), and H1t (white). Scale bars: 10 μm. **h** Quantification of MLH1 foci in mid-pachytene spermatocytes. *n*, the total number of cells analyzed from two animals per genotype. The bars represent mean ± SD. ***P < 0.001; Mann–Whitney test. **i** Representative spreads of mid-pachytene spermatocytes from WT and KI mice stained for SYCP3 (magenta), HEI10 (green), and H1t (white). Scale bars: 10 μm. **j** Quantification of HEI10 foci in mid-pachytene spermatocytes. *n*, the total number of cells analyzed from three animals per genotype. The bars represent mean ± SD. ***P < 0.001; Mann–Whitney test. **k** Representative images of the histology of ovaries from WT and *Redic1* KI female mice at the indicated ages. The primordial (green arrows), primary (yellow arrows), and secondary (magenta arrows) follicles are indicated in the ovarian sections of 5 dpp and 14 dpp mice. Scale bars, 50 μm. **l** Representative images of surface-spread oocytes from 18.5 dpc WT and *Redic1* KI mice stained for MLH1 (green) and SYCP2 (magenta). Scale bars: 10 μm.

The *Redic1* KI male mice had smaller testes and a significant reduction in the testis-to-body weight ratio when compared to those of the WT littermates (6.75 ± 0.61 × 10^−3^ in WT vs 2.80 ± 0.42 × 10^−3^ in *Redic1* KI mice; *n* = 3; *P* < 0.001; unpaired *t*-test; Fig. 3a, b). No sperm were observed in the epididymides of KI mice (Fig. 3c). Correspondingly, histological analyses showed that *Redic1* KI mice had no postmeiotic germ cells in their testes and no sperm in the epididymides, which is much distinct from the WT mice that contain numerous spermatogenic cells of different developmental stages in testes and abundant sperm in epididymides (13.27 ± 1.62 × 10^6^, *n* = 3; Fig. 3c, d). These results demonstrate that spermatogenesis in *Redic1* KI mice was arrested at the spermatocyte stage, similar to the observations in testes of the affected individuals carrying the homozygous *REDIC1* mutation c.232_233insTT.

### Spermatogenesis of *Redic1* KI mice was arrested at meiotic metaphase I due to greatly reduced bivalents

To clarify the exact substage of meiotic arrest that occurred in *Redic1* KI males, we first examined the progression of meiotic prophase I by immunostaining spermatocyte spreads for SYCP3 and γH2AX (a marker of DNA breaks). Leptotene, zygotene, pachytene, and diplotene spermatocytes were counted in both WT and KI mice, and there was no difference in the proportion of each cell type between WT and *Redic1* KI mice (Supplementary Fig. S14). These results indicate that the *Redic1* mutation has little effect on the progression of meiotic prophase I.

Next, we prepared testicular metaphase chromosome spreads and analyzed the chromosome composition in spermatogenic cells (Fig. 3e). In WT cells, the morphology of bivalent chromosomes was typical, and the number of bivalents per nucleus averaged 19.7 ± 0.5. However, in *Redic1* KI metaphase I spermatocytes, only 4.0 ± 2.6 bivalents per cell on average were observed, and the vast majority of chromosomes were presented as univalent (Fig. 3f). Furthermore, metaphase II spermatocytes and large numbers of sperm were found in WT mice, but there were no such cells in *Redic1* KI mice. Therefore, we believe that the *Redic1* mutation results in metaphase I arrest. To confirm this, we also checked the morphology of the spindles by costaining of spermatocyte smears with Tubulin and SYCP2 antibodies. In WT mice, 84.3% (70/83) of metaphase I spermatocytes have homologous chromosomes perfectly aligned to the equatorial plate and form a typical bipolar spindle. However, no such metaphase cells were seen in *Redic1* KI mice. Instead, the chromosomes were disorganized and the morphology of the spindles was abnormal in all the observed metaphase I spermatocytes (*n* = 54 cells) of *Redic1* KI mice (Supplementary Fig. S15).

### *Redic1* KI mice show reduced class I crossover-specific recombination complexes in both males and females

Given that the reduction in bivalent formation in metaphase I cells is usually caused by decreased crossover formation between homologous chromosomes in prophase^46^, we thus analyzed the late recombination nodule components MutLγ, cyclin-dependent kinase CDK2, and ubiquitin ligase HEI10 in spread pachytene spermatocytes, which are required for the formation of class I crossovers^15,16,32,35,47^. *Redic1* KI mice had a significantly fewer number of MLH1 foci (0.9 ± 1.3; *n* = 40 nuclei; Fig. 3g, h) and MLH3 foci (1.1 ± 1.8; *n* = 119 nuclei; Supplementary Fig. S16a, b) per cell than WT mice (MLH1: 22.8 ± 2.0; *n* = 61 nuclei; MLH3: 22.5 ± 2.1; *n* = 122 nuclei). Similarly, the focus number of non-telomeric CDK2, which is involved in crossover formation^47,48^, was reduced to 1.8 ± 2.2 per cell (*n* = 58), much fewer than in the WT spermatocytes (19.4 ± 2.2; *n* = 53 nuclei) at mid-pachynema, as evidenced by costaining of CDK2 with SYCP3 and H1t, a testis-specific histone 1 variant that was expressed from mid-pachytene onward, although the telomere localization of CDK2 was unaffected (Supplementary Fig. S16c, d). Moreover, HEI10 formed ~23 bright foci in each WT mid- or late pachytene spermatocyte, whereas only 2.5 ± 3.3 HEI10 foci per cell were observed in the spermatocytes of *Redic1* KI mice (Fig. 3i, j). Thus, the formation of class I crossovers is severely impaired in spermatocytes of *Redic1* KI mice.

Considering *Redic1* expression in fetal ovaries, as mentioned above, we also characterized the phenotypes of *Redic1* KI female mice. *Redic1* KI female mice did not produce any pups during the two months of crossing with WT males, and thus they are infertile. The histological analysis showed that the ovaries of 2-month-old *Redic1* KI females had no follicles, much different from the ovaries of their WT littermates, in which follicles at different developmental stages were evident (Fig. 3k). To clarify when follicle loss occurs in *Redic1* KI female mice, we examined the histology of ovaries from 5 days postpartum (dpp) and 14 dpp female mice (Fig. 3k; Supplementary Fig. S17). At 5 dpp, many primordial, primary, and secondary follicles were seen in the ovaries of control mice, while in *Redic1* KI female mice, primordial follicles were rare, and the numbers of primary and secondary follicles were reduced, suggesting that follicle formation is severely impaired after *Redic1* mutated. At 14 dpp, a large number of follicles, including primordial, primary, and secondary follicles, were observed in the ovaries of WT mice, but few follicles were present in the ovaries of *Redic1* KI mice, suggesting that follicle development is also impaired after *Redic1* mutated. Furthermore, we evaluated crossover formation in female mice by staining the spread oocytes for MLH1 and SYCP2 (Fig. 3l). Similarly, MLH1 foci in the oocytes of *Redic1* KI females (3.8 ± 2.9; *n* = 58 nuclei) were much fewer than those in the control animals (26.5 ± 4.0; *n* = 23 nuclei; Supplementary Fig. S18). Taken together, class I crossover formation is disrupted in both male and female *Redic1* KI mice.

### REDIC1 promotes meiotic DSB repair by facilitating the accumulation of ZMM proteins along the axes

**Fig. 4:**
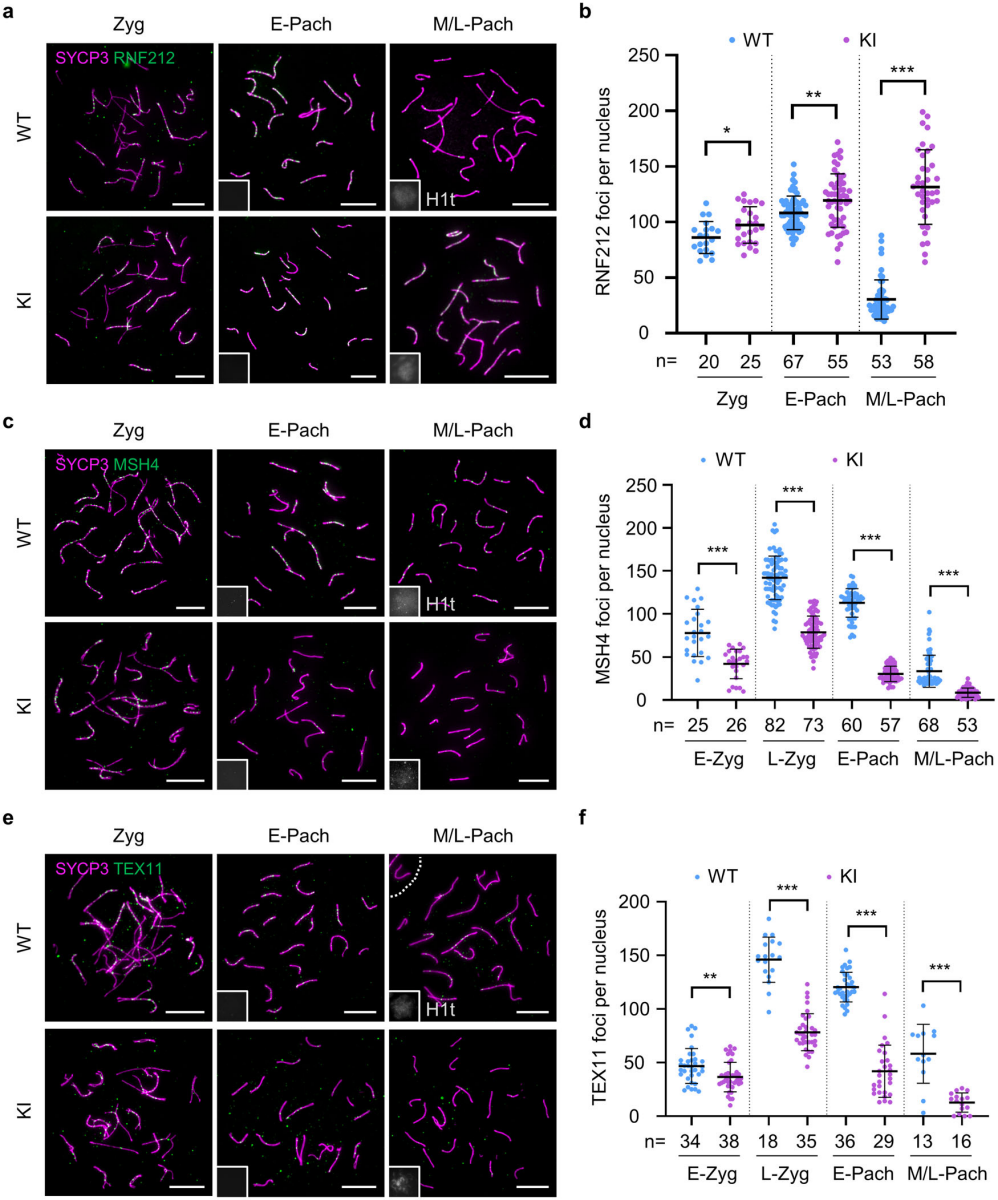
The abnormal dynamics of ZMM proteins in *Redic1* KI mice. **a** Representative images of spread spermatocytes from adult WT and *Redic1* KI mice immunostained for SYCP3 (magenta), RNF212 (green), and H1t (white, in the insets). Zygotene, early pachytene (H1t-negative), and mid- or late pachytene (H1t-positive) spermatocytes are shown. **b** Quantification of RNF212 foci in spermatocytes at the indicated substages. *n*, the total number of nuclei analyzed from two animals for each genotype. **c** Representative spread spermatocytes of WT and *Redic1* KI mice immunostained with antibodies against SYCP3 (magenta), MSH4 (green), and H1t (white, in the insets). Zygotene, early pachytene (H1t-negative), and mid/late pachytene (H1t-positive) spermatocytes are shown. **d** Quantification of MSH4 foci per cell at the indicated substages. *n*, the total number of nuclei analyzed from three animals for each genotype. **e** Representative images of spread spermatocytes from adult WT and *Redic1* KI mice immunostained for SYCP3 (magenta), TEX11 (green), and H1t (white, in the insets). Zygotene, early pachytene (H1t-negative), and mid/late pachytene (H1t-positive) spermatocytes are shown. **f** Quantification of TEX11 foci in spermatocytes at the indicated substages. *n*, the total number of nuclei analyzed from two animals for each genotype. Scale bars in **a**, **c**, and **e** indicate 10 μm. H1t (white) was used to differentiate the early and mid/late pachytene spermatocytes. The bars in **b**, **d**, and **f** indicate mean ± SD. *P* values were calculated by the Mann–Whitney test. ns, not significant; *P < 0.05; **P < 0.01; ***P < 0.001. E-Zyg, early zygotene; L-Zyg, late zygotene; E-Pach, early pachytene; M/L-Pach, mid- or late pachytene.

The RNF212 SUMO ligase plays an essential role in designating crossover sites by stabilizing the ZMM proteins MSH4 and TEX11^33^. Hence, to better understand why the number of class I crossovers decreased in *Redic1* KI mice, we examined the chromosomal localization of RNF212 in spermatocytes. As reported, RNF212 foci first appeared on the synapsed chromosome axes at the zygotene stage, and *Redic1* KI mice showed comparable numbers of RNF212 foci to the WT mice (Fig. 4a, b), indicating that the recruitment of RNF212 is not disturbed in *Redic1* mutants. At early pachytene, ~110 RNF212 foci were observed in both WT and *Redic1* KI mice. As the cells progressed to mid-pachytene (H1t-positive), RNF212 foci drastically decreased to only 30.5 ± 17.6 per cell in WT mice. However, such a decrease in RNF212 focus number was not observed in *Redic1* KI spermatocytes, which still had ~130 per cell at the mid-pachytene stage (131.5 ± 33.5, *n* = 38).

The persistence of RNF212 is often correlated with high numbers of MSH4 foci, as seen in *Cntd1*, *Hei10*, and *Prr19* knockout mice^32,49,50^. Therefore, we next examined the dynamics of MSH4 by immunostaining spermatocyte spreads (Fig. 4c, d). In WT mice, MSH4 foci were first detected at the synapsed regions of homologs at early zygotene, and the number increased to ~150 by late zygotene. Subsequently, MSH4 foci declined to ~50 at the mid-pachytene stage, consistent with the previous report^29^. Unexpectedly, fewer MSH4 foci were observed in zygotene and pachytene spermatocytes of *Redic1* KI males. The difference in the number of MSH4 foci between WT and *Redic1* KI mice was first observed at early zygonema (42.1 ± 17.2 in 26 *Redic1* KI nuclei vs 78.0 ± 27.3 in 25 WT nuclei). Although the number of MSH4 foci increased at late zygotene in *Redic1* KI spermatocytes, it remained much fewer than that in control cells (78.9 ± 18.6 in 73 *Redic1* KI nuclei vs 141.9 ± 25.3 in 82 WT nuclei). Accordingly, another ZMM protein, TEX11, showed similar dynamics to MSH4 during prophase in *Redic1* KI spermatocytes (Fig. 4e, f), indicating that REDIC1 is required for the accumulation of normal numbers of MSH4 and TEX11 foci during meiotic recombination. Considering the function of MSH4 to bind and stabilize recombination intermediates^28^, our results indicate that REDIC1 is an important factor for stabilizing recombination intermediates.

To further characterize the recombination defects in *Redic1* mutant spermatocytes, we first examined the localization of the meiosis-specific recombinase DMC1 (Supplementary Fig. S19a, b). The number of DMC1 foci in early-zygotene spermatocytes was indistinguishable between WT and *Redic1* KI mice, and averaged more than 200 foci per nucleus, suggesting that meiotic DSBs were normally formed in *Redic1* KI spermatocytes. However, the DMC1 focus number in *Redic1* KI spermatocytes at late zygotene was higher than that in the control (141.3 ± 27.8 per cell in 35 *Redic1* KI nuclei vs 110.2 ± 37.1 per cell in 41 WT nuclei), and still slightly higher in early and mid-pachytene spermatocytes (early pachytene: 62.3 ± 11.3 per cell in 37 *Redic1* KI nuclei vs 52.3 ± 10.6 per cell in 31 WT nuclei; mid-pachytene: 48.4 ± 13.4 per cell in 41 *Redic1* KI nuclei vs 37.6 ± 9.8 per cell in 31 WT nuclei). At late pachytene, DMC1 foci disappeared in both WT and *Redic1* KI spermatocytes. These kinetics of DMC1 indicate that DSB repair is delayed in *Redic1* KI spermatocytes.

In addition, RPA2 was also examined to evaluate DSB repair (Supplementary Fig. S19c, d). Compared to that in WT mice, the number of RPA2 foci in early zygotene spermatocytes of *Redic1* KI mice did not differ, indicating normal DSB formation and end-resection in the *Redic1* mutant spermatocytes. However, the RPA2 focus number in late zygotene and early pachytene spermatocytes of *Redic1* KI mice is fewer than those in the WT, which is consistent with the findings for MSH4 and TEX11, suggesting a reduced number of recombination intermediates in the *Redic1* mutant spermatocytes. Conversely, the number of RPA2 foci was elevated in mid- and late pachytene spermatocytes of *Redic1* KI mice, indicating a deficient repair of some DSBs in these cells after *Redic1* mutated.

### REDIC1 promotes the assembly of synaptonemal complexes

**Fig. 5:**
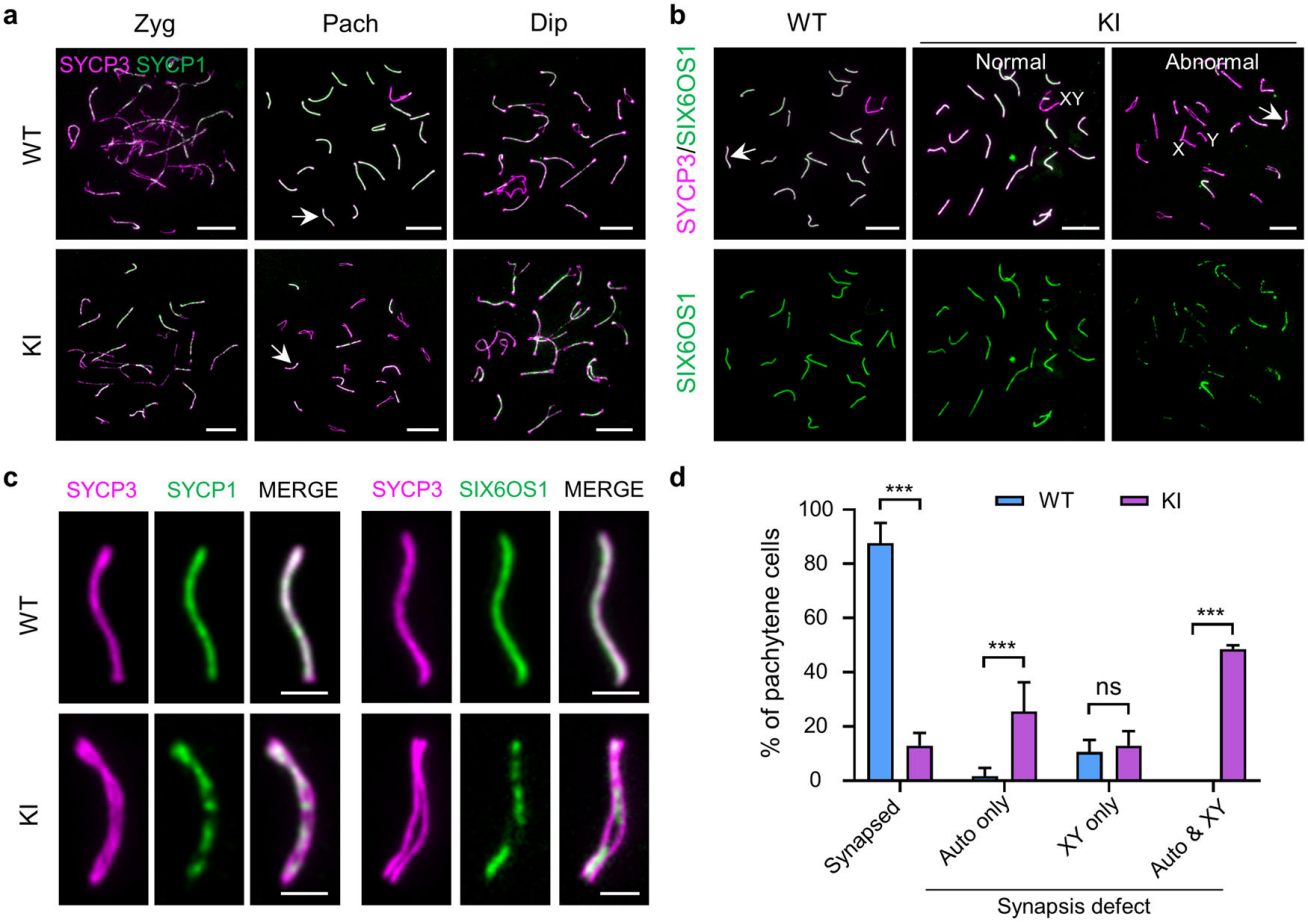
*Redic1*-deficient spermatocytes show synaptic defects. **a** Representative spread zygotene (Zyg), pachytene (Pach), and diplotene (Dip) spermatocytes of 2-month-old WT and *Redic1* KI mice immunostained for SYCP3 (magenta) and SYCP1 (green). The SCs indicated by white arrows are enlarged in panel (**c**). Scale bars, 10 μm. **b** Immunofluorescence staining of spread pachytene spermatocytes from adult WT and KI mice with antibodies against SYCP3 (magenta) and SIX6OS1 (green). For *Redic1* KI mice, both normal (left) and abnormal (right) spermatocytes are shown. The SCs indicated by white arrows are enlarged in panel (**c**). Scale bars: 10 μm. **c** Enlarged view of the chromosomes indicated by white arrows in panels (**a**) and (**b**). Scale bars: 2 μm. **d** The percentage of pachytene spermatocytes with or without synaptic defects in WT and *Redic1* KI mice. The cells were divided into four groups: with fully synapsed chromosomes; with synapsis defects only on autosomes; with synapsis defects only on XY chromosomes; and with synapsis defects on both autosomes and XY chromosomes. The experiments were performed twice on two animals for each genotype. For WT mice, 169 pachytene spermatocytes were analyzed totally; for KI mice, 196 pachytene cells were analyzed totally. The bars indicate mean ± SD. ns, not significant (*P* > 0.05); ***P < 0.001; two-way ANOVA. *Redic1* KI mice represent mice carrying the homozygous mutation c.232_233insTT in *Redic1*.

Since synapsis and homologous recombination (HR) are interdependent and closely coupled processes during meiotic prophase I^6,29^, we investigated the role of REDIC1 in chromosomal synapsis by immunostaining the spermatocyte spreads for SYCP3 and SYCP1, a transverse filament of the SC (Fig. 5a). As expected, in WT spermatocytes, the SYCP1 signals first appeared in the paired regions of homologs in early zygotene stage, extended to the entire length of the autosomal axes and the PAR of the sex chromosomes in pachytene stage, and gradually disappeared with SC disassembly in diplotene stage. In *Redic1* KI mice, typical zygotene spermatocytes were observed, but the majority of pachytene cells with short and thick lateral elements displayed discontinuous and/or weak SYCP1 signals, suggesting that synaptic defects occur in *Redic1* KI spermatocytes.

To confirm this, we stained the spermatocyte spreads with antibodies against SYCP3 and the SC central element protein SIX6OS1 (Fig. 5b). Short SIX6OS1 stretches were observed in the paired regions between homologs in zygotene cells, with no distinguishable differences among WT and *Redic1* KI animals. However, in *Redic1* mutant pachytene spermatocytes, which have similar lateral element length and thickness as well as sex chromosome morphology to the WT pachytene cells, incomplete synapsis of several autosomes and asynapsis of XY chromosomes were observed. Similar to the observation in SYCP1 staining spermatocytes, discontinuous SIX6OS1 signals are evident in the pachytene spermatocytes of *Redic1* KI mice (Fig. 5c). Remarkably, in WT mice, the autosomes of all pachytene spermatocytes achieved complete synapsis, and 10.62 ± 4.43% of cells exhibited sex chromosome separation, which is consistent with our previous report^9^. However, in *Redic1* KI mice, although 12.98% of pachytene spermatocytes presented fully synapsed chromosomes, the remaining 87.12% showed variable synaptic abnormalities. Among them, 25.56% of cells showed synapsed sex chromosomes but with at least one pair of incompletely synapsed autosomes, 12.92% of cells showed fully synapsed autosomes but with asynapsed sex chromosomes, and 48.54% contained synapsis defects on both autosomal and sex chromosomes (Fig. 5d). Thus, these findings indicate that REDIC1 plays an essential role in complete chromosomal synapsis.

### Men with the homozygous *C12orf40* mutation reproduce the recombination failure and synaptic defects of *Redic1* KI mice

**Fig. 6:**
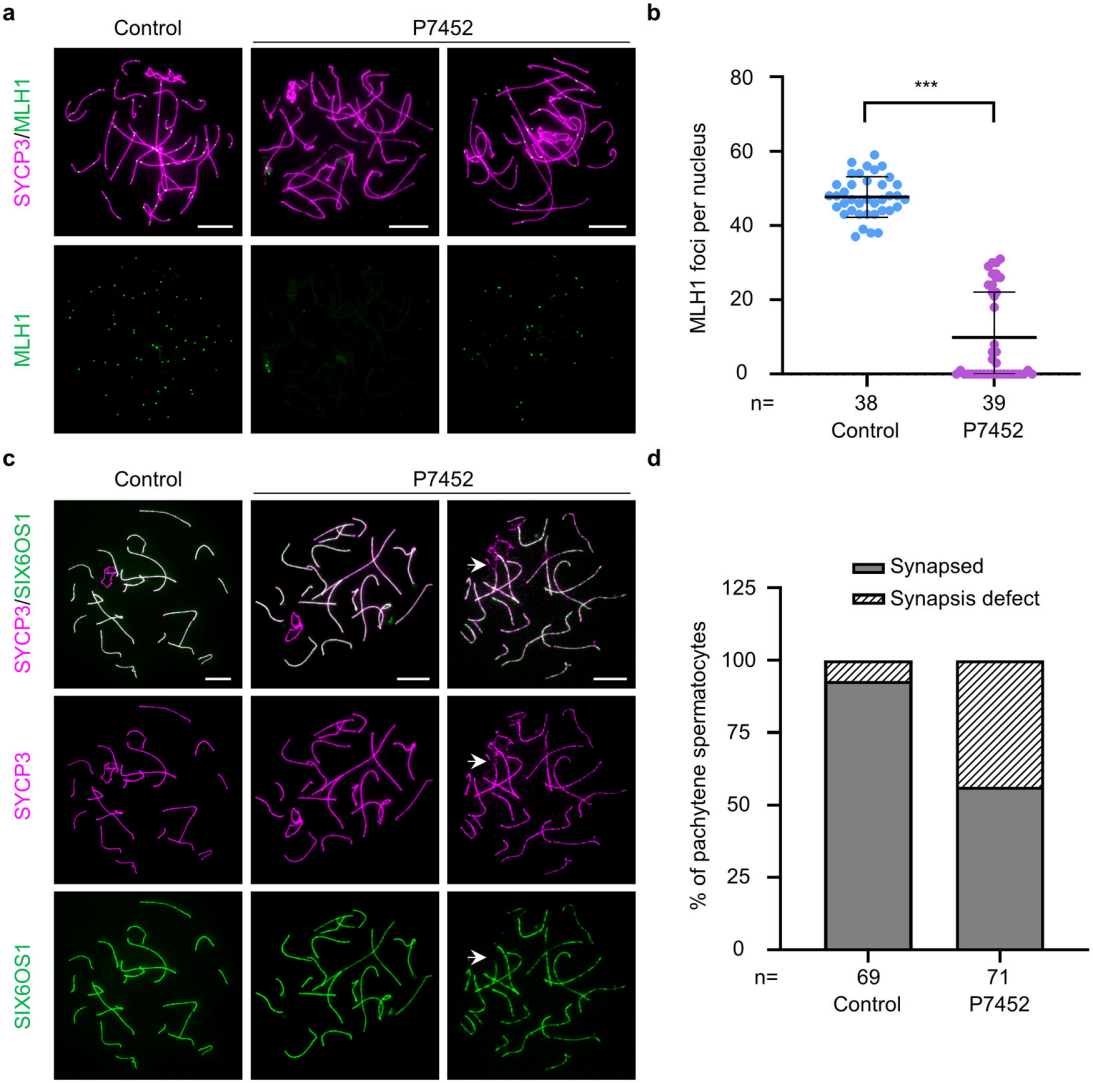
The man carrying the *C12orf40* homozygous mutation also presented reduced meiotic crossovers and synaptic defects. **a** Representative images of surface-spread spermatocytes from the control and individual P7452 immunostained for SYCP3 (magenta) and MLH1 (green). A mid-pachytene spermatocyte with no MLH1 foci and a mid-pachytene spermatocyte with a reduced number of MLH1 foci in individual P7452 are shown. The spermatocytes from a man who was diagnosed with obstructive azoospermia were used as the control. Scale bars: 10 μm. **b** Quantification of MLH1 foci per spermatocyte in the control and individual P7452. *n*, the number of cells scored. The bars indicate mean ± SD. ***P < 0.001; Mann–Whitney test. **c** Immunofluorescence staining of pachytene spermatocytes from individual P7452 and the control (the same man used in **a** and **b**) with antibodies against SYCP3 (magenta) and SIX6OS1 (green). A pachytene spermatocyte with normal synapsis and a pachytene cell with synapsis defect on one pair of autosomes (indicated by the white arrow) are shown. Scale bars: 10 μm. **d** The proportion of normal (with all chromosomes synapsed) and abnormal (containing at least one chromosome with synapsis defect) cells in panel (**c**). *n*, the total number of pachytene cells scored.

Next, we wondered whether the affected men with the homozygous *C12orf40* mutation c.232_233insTT exhibit phenotypes similar to those observed in *Redic1* KI mice. Crossover formation was examined by immunostaining human spermatocyte spreads for SYCP3 and the class I crossover marker MLH1, as we did for the mouse model. In the control, the number of MLH1 foci averaged ~48 per nucleus (47.7 ± 5.5; *n* = 38 nuclei), which was consistent with our previous report^51^. Conversely, the number of MLH1 foci was significantly reduced in the pachytene spermatocytes of the affected individual P7452 (9.9 ± 12.2; *n* = 39 nuclei; Fig. 6a, b), indicating that C12ORF40 plays a conserved role in the formation of class I crossovers in humans.

Subsequently, we performed SYCP3 and SIX6OS1 staining on spermatocyte spreads of the control and individual P7452 to evaluate chromosomal synapsis (Fig. 6c). The behavior of chromosomes in leptotene and zygotene spermatocytes of individual P7452 was normal. In agreement with our previous report^52^, in the control, as the cells proceeded to the pachytene stage, the SIX6OS1 signals extended to the entire chromosomal axes of autosomes, and more than 92% of pachytene spermatocytes presented fully synapsed homologs. However, in all the 71 pachytene cells analyzed from individual P7452, the proportion of cells with fully synapsed homologs was 56.34%, and the remaining 43.66% of cells contained autosomes with forked or bubbled regions devoid of SIX6OS1 signals (Fig. 6d), indicating that the identified *C12orf40* mutation can cause chromosomal synaptic defects in humans. Taken together, the affected individuals with a homozygous *C12orf40* frameshift mutation resemble the phenotypes observed in *Redic1* KI mice.

### The mutation in C12ORF40/REDIC1 largely impairs its ability to bind branched recombination intermediates

**Fig. 7:**
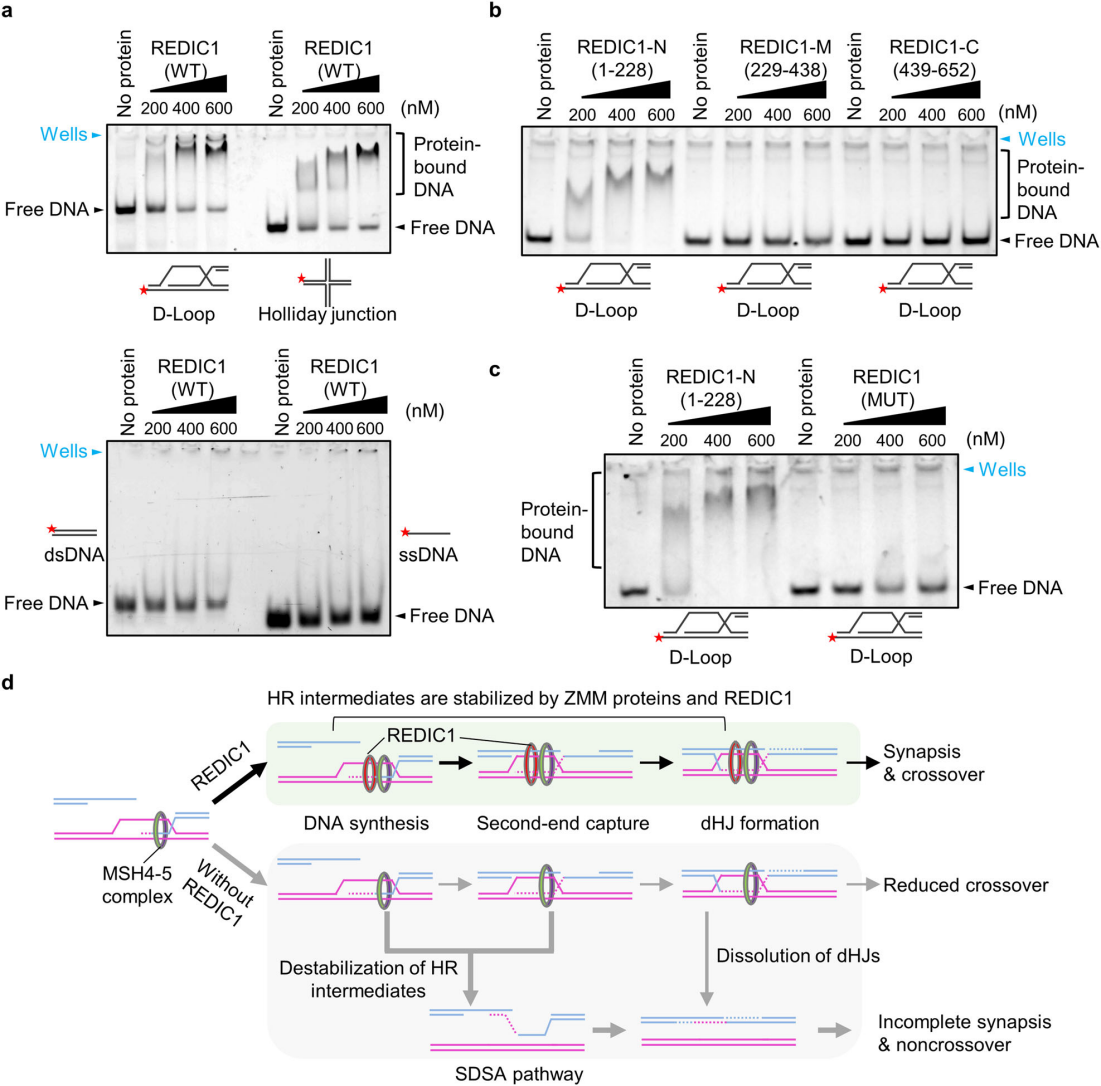
The frameshift mutation in C12ORF40/REDIC1 largely impairs its ability to bind branched recombination intermediates. **a** EMSA with purified REDIC1 protein (WT) and the indicated DNA substrates. The experiments were repeated three times with similar results. For each group, 100 nmol of 5-FAM-labeled DNA substrates were used. **b** EMSA with purified REDIC1 N-fragment (1–228 aa), M-fragment (229–438 aa), or C-fragment (439–652 aa) and D-loop. The experiments were repeated twice with similar results. For each group, 100 nmol of 5-FAM-labeled DNA substrates were used. **c** EMSA with purified REDIC1 N-fragment (1–228 aa) or mutant (p.Met78Ilefs*2) and D-loop. **d** Schematic diagram showing the proposed function of REDIC1 in meiotic recombination. Following the strand invasion, the nascent D-loop is first bound and stabilized by the MSH4–5 complex. The binding of REDIC1 not only promotes chromosome synapsis but also further stabilizes HR intermediates, allowing some DSBs to be processed into dHJs, ultimately leading to the formation of crossover. In the absence of REDIC1, some recombination intermediates are destabilized, thereby forming a reduced number of crossovers via the DSBR pathway for a few DSBs; other DSBs may form non-crossovers via the SDSA pathway or the dissolution of dHJs. In addition, the destabilization of recombination intermediates can lead to synaptic defects.

Finally, we tried to understand how REDIC1 stabilizes recombination intermediates. Although the online tools did not predict known structural domains, based on its punctate localization after strand invasion and its function in crossover formation, we speculated that REDIC1 could be a potential DNA-binding protein similar to MutSγ, which has been shown to bind branched recombination intermediates^53^. Thus, we purified the full-length REDIC1 protein and performed electrophoretic mobility shift assays (EMSAs) as reported^53^. The EMSA results showed that no or very few protein-bound DNA complexes were observed after incubation of the WT REDIC1 protein with ssDNA or paired double-stranded DNA (dsDNA), whereas the protein-bound DNA complexes were easily seen after incubation with DNAs containing a D-loop or Holliday junction structures (Fig. 7a; Supplementary Fig. S20a), indicating that C12ORF40/REDIC1 binds to branched DNA molecules.

To map the region responsible for DNA binding, we divided the full-length REDIC1 protein into N-fragment (1–228 aa), M-fragment (229–438 aa), and C-fragment (439–652 aa), and performed EMSA with the DNAs containing a D-loop as the substrates (Fig. 7b; Supplementary Fig. S20b). Interestingly, the protein-bound DNA complex was detected only after incubation of the REDIC1-N fragment with the DNAs containing a D-loop, suggesting that the N-terminus of REDIC1 is responsible for its binding to the branched DNA molecules. Since the identified frameshift mutation was predicted to produce a truncated REDIC1 protein (p.Met78Ilefs*2), we thus wondered whether this truncated protein binds to the DNAs containing a D-loop. In contrast to the REDIC1-N fragment, there was little protein-bound DNA observed after incubation with equimolar amounts of truncated REDIC1 protein with DNAs containing a D-loop (Fig. 7c; Supplementary Fig. S20c), indicating that the identified frameshift mutation largely impairs the DNA-binding ability.

Based on the results, we propose a model for the function of C12ORF40/REDIC1 (Fig. 7d). During meiotic recombination, the newly formed D-loop is first bound and stabilized by ZMM proteins, including MSH4/MSH5 and TEX11. The binding of REDIC1 not only promotes chromosome synapsis but also enhances the stabilization of recombination intermediates, which facilitates their undergoing DNA synthesis, D-loop migration, second-end capture, and being processed to form dHJs and eventually crossovers. However, the identified mutation in C12ORF40/REDIC1 results in the destabilization of some recombination intermediates, which ultimately leads to the formation of a reduced number of crossovers via the DSBR pathway for a few DSBs; other DSBs may form noncrossovers via the SDSA pathway or the dissolution of dHJs. In addition, the destabilization of recombination intermediates could cause synaptic defects.

## Discussion

Defects in crossover formation can result in meiotic arrest or aneuploidy, ultimately damaging human fertility^2,54,55^. In this study, for the first time, we identified a homozygous frameshift mutation c.232_233insTT in *C12orf40*, a previously uncharacterized gene, from two genetically unrelated azoospermic men with meiotic arrest and revealed its essential role in meiotic crossover formation. Through functional analysis of *Redic1* KI mutation mice, we demonstrated that this mutation disrupts the ability to bind branched recombination intermediates, leading to a drastic reduction in crossovers and an increase in synaptic defects in both males and females. Consistently, these phenotypes were also observed in the spermatocytes of patients carrying this *C12orf40* homozygous mutation. Thus, we identified C12ORF40/REDIC1 as a novel recombination protein essential for meiotic crossover formation and confirmed that the loss of function of this gene could result in infertility in both humans and mice.

In mammalian meiotic cells, at least two types of crossovers have been reported. The class I crossovers failed to form in *Mlh3*^–/–^ and *Mlh1*^–/–^ male mice, but 1–3 bivalents remained in the metaphase I spermatocytes^16,56^, which are thought to be contributed by the class II crossovers. In our *Redic1* KI male mice, the MLH1 focus number averaged 1 and the number of bivalents averaged 4. The difference between them is ~3, which is close to the reported number of class II crossovers. Thus, we think that REDIC1 plays a role in class I crossover formation.

The *Redic1* frameshift mutation resulted in not only reduced crossovers but also synaptic defects. This reminds us of the phenotypes of *Tex11* and *Hfm1* knockout mice. The average number of MLH1 foci per cell was reduced to ~12 in *Tex11*^-/Y^ spermatocytes and 0.8 in *Hfm1*-deficient spermatocytes^5,6^, whereas in *Redic1* KI mice, the number of MLH1 foci averaged 0.9 per spermatocyte, much less than the ~24 in WT mice, suggesting that the phenotype of *Redic1* KI mice was close to that of *Hfm1*^–/–^ mice. Notably, the proportion of pachytene cells with synapsis defects was only 38% in *Tex11*^–/Y^ mice and 46.2% in *Hfm1*^–/–^ mice^5,6^, while it was over 80% in *Redic1* KI mice. Thus, the phenotype of *Redic1* KI mice was more severe than that of *Hfm1*^–/–^ mice, suggesting that REDIC1 likely acts earlier than HFM1 or in the same complex during meiotic recombination. In addition, the phenotype of *Redic1* KI mice was milder than that of *Msh4*^–/–^ mice, in which meiotic DSB repair was blocked, homologous chromosomes failed to synapse, and meiosis was arrested at the zygotene-like stage^29^, suggesting that REDIC1 functions later than MSH4 or REDIC1 is not as important as MSH4 in meiotic crossover formation. Taken together, the time window for REDIC1 action coincides with the transition of recombination intermediates to crossovers. Furthermore, considering that REDIC1 is a recombination protein that colocalizes with MSH4 and interacts with its partner MSH5 and with TEX11, and that the number of MLH3 foci in *Redic1* mutant mid-pachytene spermatocytes with fully paired autosomes (“WT” like cells) is much fewer than that of controls, we think the synaptic defects in *Redic1* KI mice are more likely the consequence of recombination defects.

During meiosis, programmed DSBs are repaired by HR, which involves the generation of different recombination intermediates between paternal and maternal chromosomes^57^. The stabilization of these recombination intermediates is not only essential for the proper alignment and synapsis of homologous chromosomes but also for the formation of crossovers^57,58^. Based on our data, we believe that REDIC1 plays a role in stabilizing recombination intermediates. First, our cytological evidence indicates that REDIC1 is punctately localized to paired regions of homologous chromosomes; second, genetic evidence suggests that chromosome axis localization of REDIC1 is dependent on strand invasion rather than chromosomal synapsis; third, our biochemical experiments show that REDIC1 directly binds branched recombination intermediates such as D-loops and Holliday junctions. In addition, phenotypic analysis of *Redic1* KI mice showed that the focus numbers of MSH4 and TEX11, two well-known proteins localized to recombination intermediates^5,28,53^, were significantly reduced, indicative of unstable recombination intermediates. Therefore, we conclude that C12ORF40/REDIC1 facilitates crossover formation by stabilizing recombination intermediates.

Interestingly, the recruitment of RNF212 in our *Redic1* KI mice was unaffected at the zygotene stage, but its dissociation from the chromosomal axes during the pachytene stage was disturbed. This phenotype is reminiscent of the observations in *Cntd1-*, *Hei10-*, and *Prr19*-deficient mice^32,49,50^, in which RNF212 and MSH4 recruitment to the chromosomal axes was not affected, but their focus numbers failed to decrease from the early pachytene to mid-pachytene stage, and they did not dissociate from the axes until the cells entered the diplotene stage. RNF212 localization to the chromosome axis is largely dependent on synapsis, especially the central elements of the SCs, and its subsequent elimination requires HEI10-mediated ubiquitination^32,33^. The retention of RNF212 in mid/late pachytene spermatocytes of *Redic1* KI mice may be due to the insufficient recruitment of HEI10. Consistent with this, the number of HEI10 foci was greatly reduced in our *Redic1* KI mice, suggesting that REDIC1 may have a late role in the turnover of RNF212, possibly by recruiting HEI10 or acting synergistically, which is crucial for crossover designation. In addition, we cannot exclude the possibility that the recombination process did not progress to the formation of the HEI10 foci in *Redic1* KI mice. In this case, although REDIC1 is colocalized with MLH1 at crossover sites and deficiency of *Redic1* reduced crossover formation, whether REDIC1 is directly involved in crossover formation needs more experimental evidence. Notably, the initial recruitment of MSH4 in *Redic1* KI mice was significantly less than that in WT mice and then remained at lower levels in subsequent substages, which is different from the observations in *Cntd1-*, *Hei10-*, and *Prr19*-deficient spermatocytes. This could be due to the instability of MSH4 itself or the instability of the recombination intermediates leading to the subsequent precocious collapse. Given that TEX11 exhibited similar dynamics to that of MSH4 and that REDIC1 can bind directly to branched DNA molecules, we prefer that the reduced number of ZMM proteins on the chromosome axes is a consequence of the destabilization of the recombination intermediates.

Meiotic recombination not only ensures the correct segregation of homologous chromosomes but is also a source of genetic diversity. DMC1 is a meiosis-specific recombinase that, together with RAD51, mediates the strand invasion^59–61^. In *Redic1* KI mice, the numbers of DMC1 foci in late zygotene and pachytene spermatocytes are higher compared to the WT mice, suggesting a delayed DSB repair, which is similar to the observations in *Zip4/Tex11* and *Hfm1* mutant mice^6,31^. One explanation is that REDIC1 may be involved in the release of DMC1 from the recombination intermediates by itself or by recruiting other factors required for the turnover of RAD51/DMC1 filaments, while this function was disturbed to some extent, thereby leading to an elevated DMC1 focus number in the spermatocytes of *Redic1* KI mice. Another possible explanation is that the second-end capture is delayed or fails at some DSB sites that enter the DSBR pathway, resulting in more DMC1 foci retained on the second end of those DSBs in the spermatocytes of *Redic1* KI mice.

In this study, we reveal the important role of C12ORF40/REDIC1 in stabilizing recombination intermediates during meiotic crossover formation and verify that its loss-of-function mutation leads to spermatogenic failure in both humans and mice. Our current study not only provides new insights into meiotic recombination but also provides a prospective molecular target for the clinical diagnosis and treatment of infertility.

## Materials and methods

### Participants

The two men affected by nonobstructive azoospermia were recruited from Nanjing, China. They were neither siblings nor relatives. Written informed consent was obtained from all the participants. Semen analyses were performed following the laboratory manual of the World Health Organization^62^. The reproductive hormones and karyotypes were analyzed by local clinicians. This study was conducted in accordance with the Declaration of Helsinki and approved by the Ethical Committee of the University of Science and Technology of China (USTC) with approval number 2019-KY-168.

### Genetic and bioinformatic analyses

Genomic DNA was extracted from the peripheral blood cells of the two infertile men using the FlexiGene DNA Kit (Qiagen, 51206). Exons were captured using the Agilent SureSelect XT 50 Mb Exon Capture Kit, and high-throughput sequencing was performed on an Illumina HiSeq 2000 platform. The reads were aligned to the human genome reference assembly with the Burrows-Wheeler Aligner using default parameters. The variants were called using the Genome Analysis Toolkit Unified Genotyper and filtered according to the criteria set in the flow chart of Supplementary Fig. S1. The candidate variant was validated using Sanger sequencing.

### Histological analysis

For the testes and epididymides, tissues were fixed in Bouin’s fixative solution overnight. After dehydration through gradient ethanol, the tissues were embedded in the paraffin and cut into 5 μm-thick sections. The slides were deparaffinized, rehydrated, and then sequentially stained with hematoxylin and eosin. For the ovaries, fresh tissues were fixed in a 4% formaldehyde solution. The paraffin-embedded ovaries were sectioned on slides at an 8-μm thickness and stained with hematoxylin. The images were captured either via a Nikon ECLIPSE 80i microscope with a DS-Ri1 camera and processed with NIS-elements BR software or by an Olympus BX53 microscope (Olympus, Tokyo, Japan) equipped with an SC-180 camera and processed with Olympus cellSens software.

### Antibody generation

The REDIC1 fragment (500–652 aa) was used as an antigen to immunize rabbits. Polyclonal antibodies were purified from the serum using Protein A/G spin columns (GE Healthcare) by Dia-an Biotech (Wuhan, China) Co., Ltd. The specificity of the antibodies was verified by western blot and immunofluorescence staining in WT and *Redic1* KI mice.

### Mice

*Redic1* KI mice mimicking the *REDIC1* mutation c.232_233insTT identified in our patients were generated using CRISPR/Cas9 genome editing technology as we reported^63^. Single-guide RNAs (sgRNAs) were designed using the CRISPOR online program (http://crispor.tefor.net/)^64^. The sgRNA sequence, single-stranded oligonucleotides, and genotyping primer sequences are shown in Supplementary Table S2. Cas9 mRNA, sgRNAs, and oligonucleotides were coinjected into the zygotes of C57BL/6 mice, followed by embryo transfer into pseudopregnant ICR females. Genomic DNA was extracted from the toes of founder mice, and genotyping was performed by polymerase chain reaction and Sanger sequencing. The founder mice carrying the target KI mutation were crossed with WT C57BL/6 mice to obtain offspring. *Six6os1*^–/–^ mice were also generated by CRISPR/Cas9 technology to target exon 4, and 5-bp nucleotides were deleted. *Top6bl*^–/–^ mice^65^ were maintained in our laboratory. *Dmc1*^–/–^ mice^42^ were obtained from the Jackson Laboratory (JAX: 008608). *Redic1*-Flag/Myc mice with a tandem 3× Flag-3× Myc tag sequence inserted between *Redic1*’s last coding amino acid and the stop codon were produced by the Laboratory Animal Center of the USTC using CRISPR/Cas9 technology. All animal studies were approved by the Institutional Animal Care and Use Committee of the USTC (USTCACUC25090122076) and performed following the committee guidelines.

### Nuclear surface spread preparation and immunofluorescence

Spermatocyte spreads were prepared as we previously described^65,66^. In brief, the seminiferous tubules were treated with hypotonic extraction buffer for 30 min, and then germ cells were squeezed out in one drop of 100 mM sucrose solution and spread on slides with 1% PFA containing 0.15% Triton X-100. The slides were placed in a humidified chamber for at least 2 h and air-dried. Oocyte spreads were prepared from ovaries of 16.5–18.5 dpc female mice as previously reported^9,67^. Immunofluorescence staining was carried out as we previously described^52^. The primary and secondary antibodies used and their dilutions are shown in Supplementary Table S3. Conventional fluorescence images were captured with an Olympus BX53 microscope (Olympus, Tokyo, Japan) with a scientific complementary metal-oxide-semiconductor camera (Prime BSI, Teledyne Photometrics Inc., USA) and processed with the Olympus cellSens software. Super-resolution images were captured using structured illumination microscopy (Nikon, N-SIM) equipped with a 100× oil-immersion objective lens (SR Apo TIRF 100×, NA 1.49) and a CCD camera (Andor, DU-897, X-11459), and images were processed using the NIS-Elements software.

### Staging of meiotic prophase

Nuclear spreads were staged based on the development of axial/lateral elements (AE/LEs, labeled by SYCP3 or SYCP2) and chromosome synapsis (marked by SYCP1 or SIX6OS1 signals), as previously described^68^. Leptotene: short stretches of AEs without synapsis; early zygotene: relatively long and incomplete LEs with visible SIX6OS1 threads; late zygotene: intact LEs but incomplete chromosome synapsis; early pachytene: all autosomes have synapsed, and thickness of the synapsed axes is uniform, synapsed PAR is long (length is > 3× the widths); mid-pachytene: the morphology of the synaptonemal complex is similar to that in early pachytene, synapsed PAR is short (length is < 3× the widths), and the X chromosome also becomes shorter than that in early pachytene; late pachytene: the ends of synapsed autosomes are thicker than the interstitial regions, synapsed PAR is very short, dot-like, or with a small gap between them; diplotene: central elements start to disassemble, but the LEs remain intact, the X chromosome is relatively long and curved. To distinguish the substages of pachytene more precisely, we used histone H1t in combination with SYCP3 staining. A field containing early pachytene and mid-pachytene spermatocytes (based on the length of synapsed PAR) was selected to determine H1t exposure. Early pachytene: no or very weak H1t staining; mid-pachytene: H1t staining is weak to intermediate; late pachytene: H1t staining is intermediate to strong. For staging of spermatocytes in *Redic1* KI mice, the same criteria as those used for WT mice were applied for the leptotene and early zygotene. Due to synaptic defects in spermatocytes of *Redic1* mutant mice, subsequent substages were classified based on the length and thickness of the lateral elements of SCs and morphology of the sex chromosomes in combination with H1t staining.

### Diakinesis/metaphase I chromosome spreading preparation

Chromosome spreads were prepared as previously described^63^. Cells on the slides were stained with Giemsa (Solarbio, G1010) according to the manufacturer’s instructions. Images were captured using a Nikon ECLIPSE 80i microscope (Nikon) equipped with a DS-Ri1 camera and analyzed with NIS-elements BR software (Nikon).

### Purification of REDIC1 protein

The WT and mutant REDIC1 coding sequences were N-terminally fused with the GST tag and C-terminally fused with the Flag tag. The primers used for the construction of plasmids are listed in Supplementary Table S4. The plasmids were transformed into *Escherichia coli* cells, and the bacteria were cultured in the 2× YT medium containing 0.7 mM IPTG at 30 °C for 5 h. Cells were resuspended in lysis buffer (20 mM Tris-HCl pH 8.0, 150 mM NaCl, 0.5% Triton X-100, 10% glycerol) containing a protease inhibitor cocktail (Sigma, P8340) and homogenized by sonication. The fusion proteins in the supernatants after centrifugation were first purified using glutathione sepharose 4 fast flow (Cytiva, 17513201), followed by a second round of affinity purification with the anti-Flag resin (Genscript, L00425), according to the manufacturer’s instructions. The purity of the proteins was assessed by SDS-PAGE combined with Coomassie brilliant blue staining. The protein concentration was determined by the Bradford method.

### EMSA

The DNA binding reactions were performed as described with minor modifications^53^. The DNA substrates, including ssDNA, dsDNA, Holliday Junctions, and D-loops, were labeled with 5-FAM, and the sequences can be found in Supplementary Table S5. In brief, the specified concentrations of proteins and 100 nM DNA substrates were assembled in binding buffer (25 mM HEPES pH 7.8, 5 mM MgCl_2_, 5% glycerol, 1 mM DTT, 50 μg/mL BSA) and incubated on ice for 15 min, followed by the addition of loading dye (50% glycerol, 0.01% bromophenol blue). The products were separated on a 6% native polyacrylamide gel (29:1 acrylamide-bisacrylamide, Sangon) in 0.5× Tris-borate-EDTA buffer on ice. The gels were scanned by an ImageQuant LAS4000 (GE Healthcare).

### Statistical analysis

Statistical analyses were conducted using GraphPad Prism 8.0 software. All tests and *P* values are described in the figure legends and/or the main text. *P* values of less than 0.05 were considered statistically significant.

### Supplementary information

Supplementary Information

## Data Availability

The *REDIC1* variant reported in this study has been submitted to ClinVar with accession number SCV002600888. The WES datasets supporting the current study have not been deposited in a public repository because of privacy and ethical restrictions but are available from the corresponding author Q.S. on reasonable request. The authors declare that the main data supporting the findings of this study are available within the article and its Supplementary information files.
