# Supplementary material for: A novel recombination protein C12ORF40/REDIC1 is required for meiotic crossover formation
Source: Cell Discov. 2023 Aug 23;9:88. doi: 10.1038/s41421-023-00577-5 (PMC10447524; doi:10.1038/s41421-023-00577-5)
Supplement: Supplementary file 1 — Supplementary Information [file 41421_2023_577_MOESM1_ESM.pdf]

A novel recombination protein C12ORF40/REDIC1 is required for meiotic crossover formation

Suixing Fan[1#], Yuewen Wang[1#], Hanwei Jiang[1#], Xiaohua Jiang[1], Jianteng Zhou[1], Yuying Jiao[1], Jingwei Ye[1], Zishuo Xu[1], Yue Wang[1], Xuefeng Xie[1], Huan Zhang[1], Yang Li[1], Wei Liu[1], Xiangjun Zhang[1], Hui Ma[1], Baolu Shi[1], Yuanwei Zhang[1], Muhammad Zubair[1], Wasim Shah[1], Zhipeng Xu[2*], Bo Xu[1*] & Qinghua Shi[1*]

Supplementary Information

Supplementary Figures

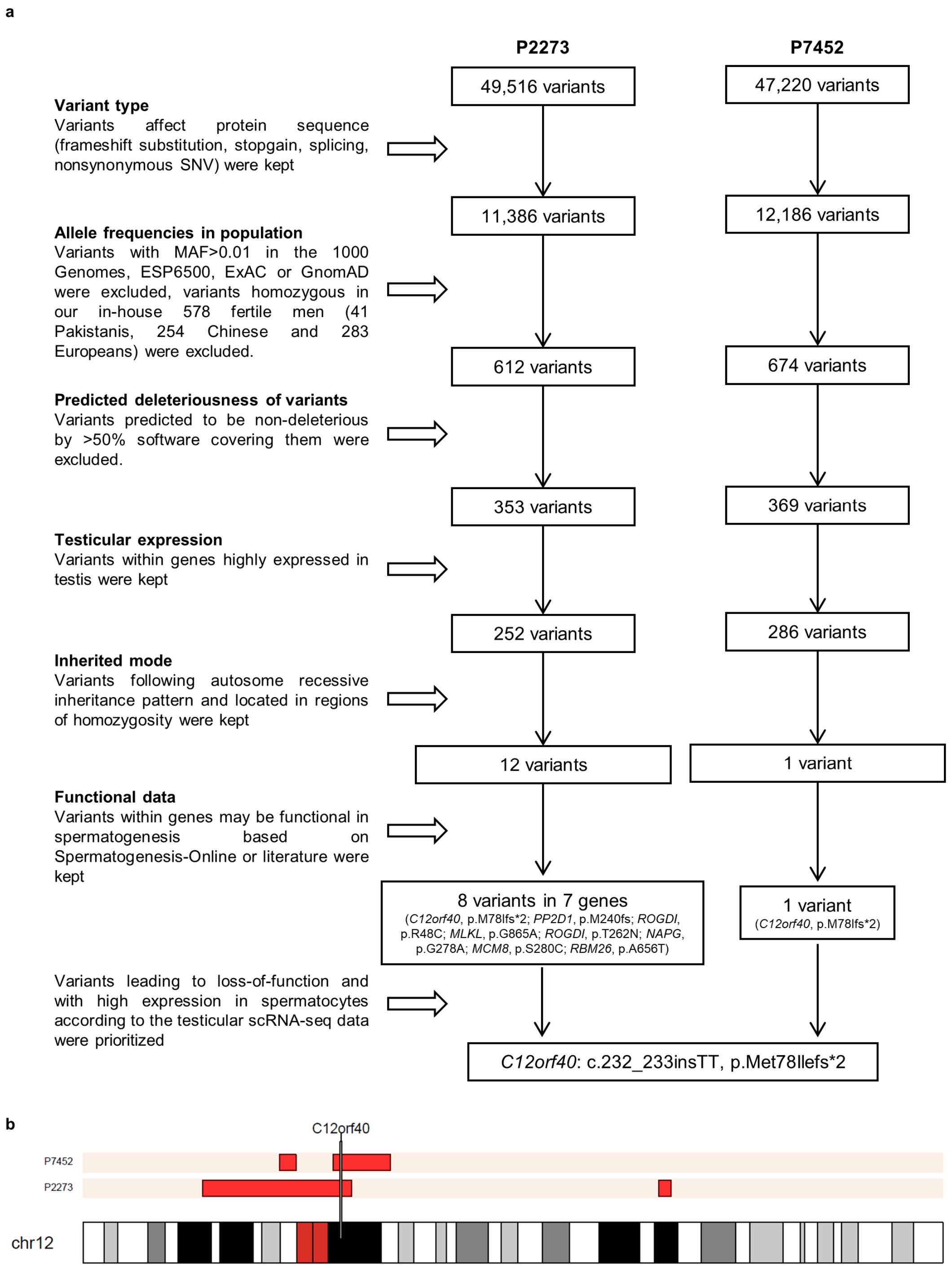

Supplementary Fig. S1 The workflow for whole-exome sequencing data analysis. **a** Bioinformatic analysis and filtering of variants. The filter criteria used are shown on the left. **b** Homozygosity mapping analysis for individuals carrying the *C12orf40* mutation.

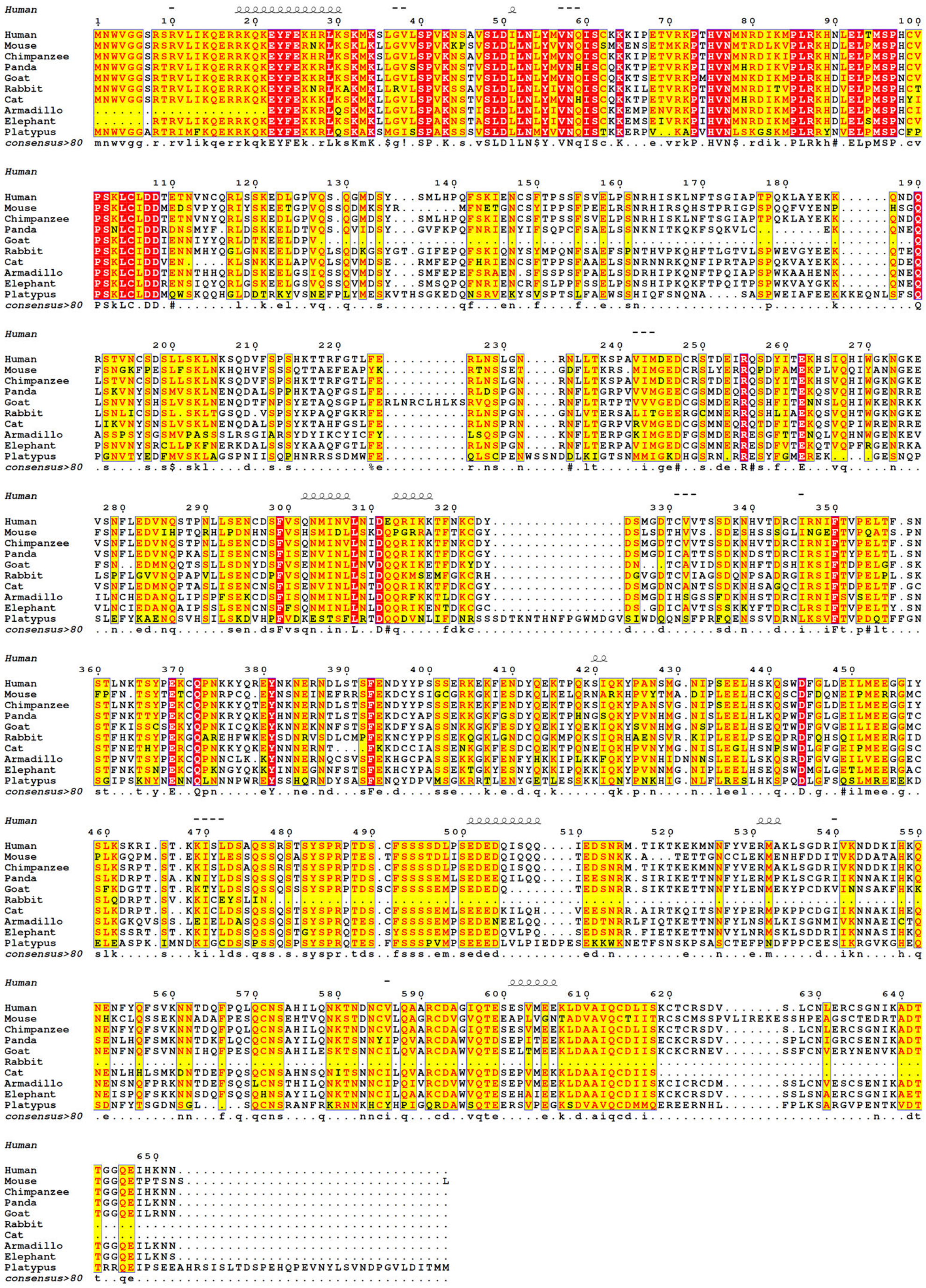

Supplementary Fig. S2 Protein sequence alignment of C12ORF40 orthologs from different species. The protein sequences of human C12ORF40 and its counterparts in other species are from the Ensembl database. Multiple sequence alignment was performed using the T-Coffee web server[1], and the image was generated by the ESPript 3.0 server[2]. Fully conserved amino acid residues are depicted in white on a red background, whereas partially conserved residues (>80%) are in red within yellow boxes. The secondary structures are predicted by the NPS@ server and shown above the alignment[3].

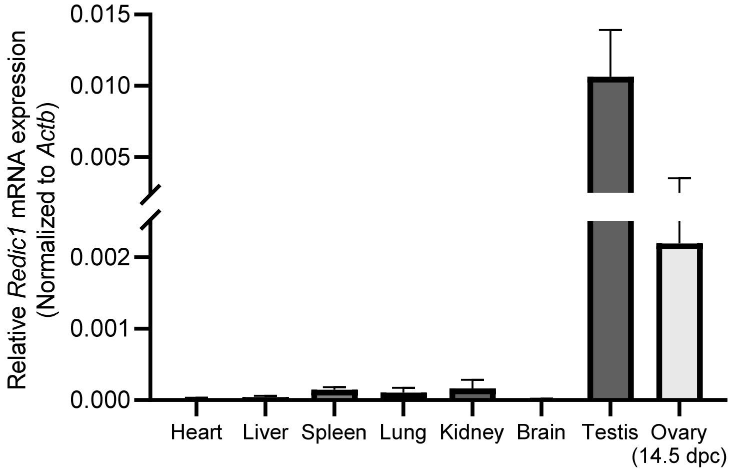

Supplementary Fig. S3 RT–qPCR analysis of *Redic1* expression in multiple mouse tissues. The heart, liver, spleen, lung, kidney, brain, and testis tissues are from 2-month-old male mice. The fetal ovaries are from 14.5 dpc female mice. *Actb* served as the reference. The experiments were performed in triplicate and the data represent mean ± SD.

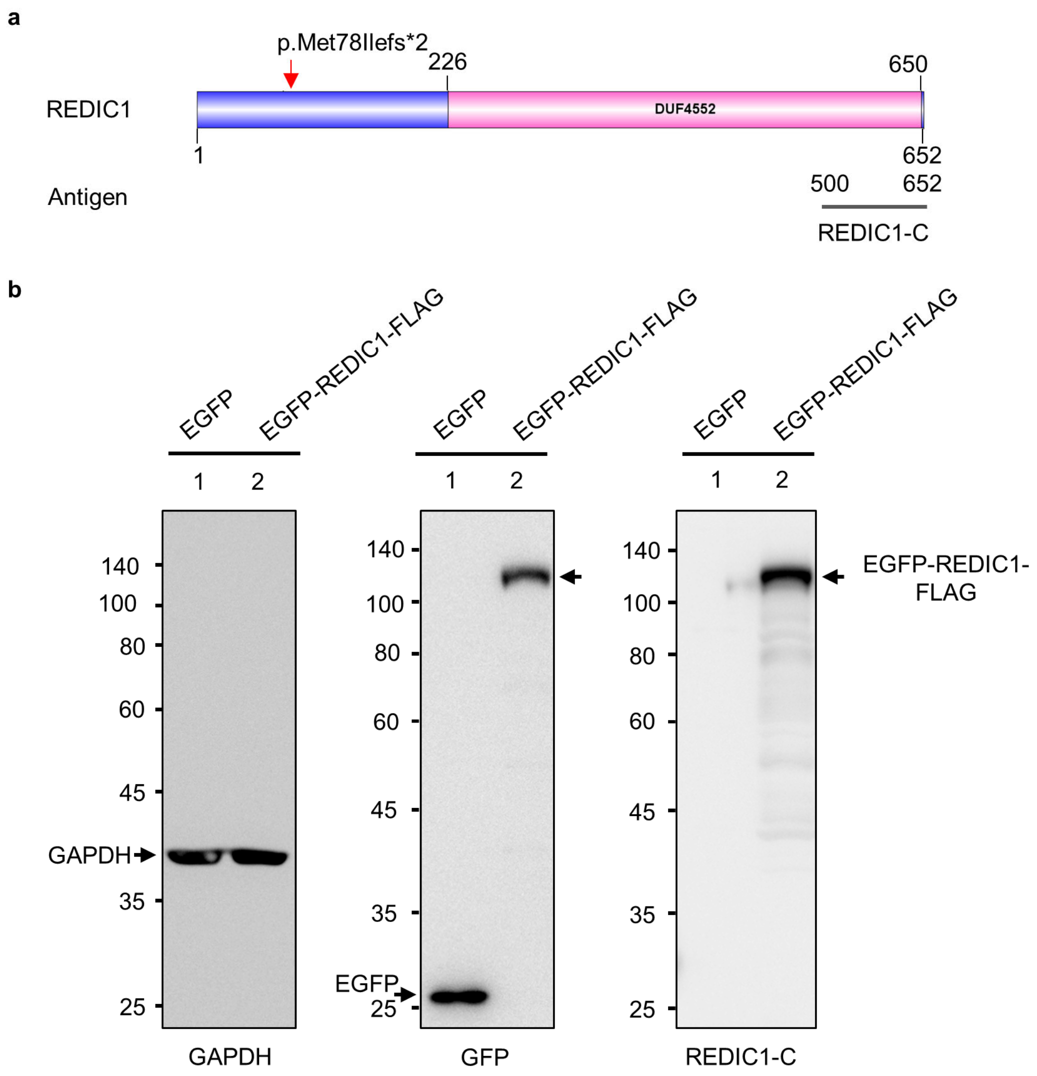

Supplementary Fig. S4 Validation of REDIC1 antibodies. a Antigen information of mouse REDIC1 antibody. A schematic diagram of mouse REDIC1 protein composition was drawn based on the prediction results of the SMART online tool[4]. **b** Western blot of lysates from in vitro overexpressed cells using REDIC1 antibody. GAPDH served as the internal control. EGFP was used as a negative control.

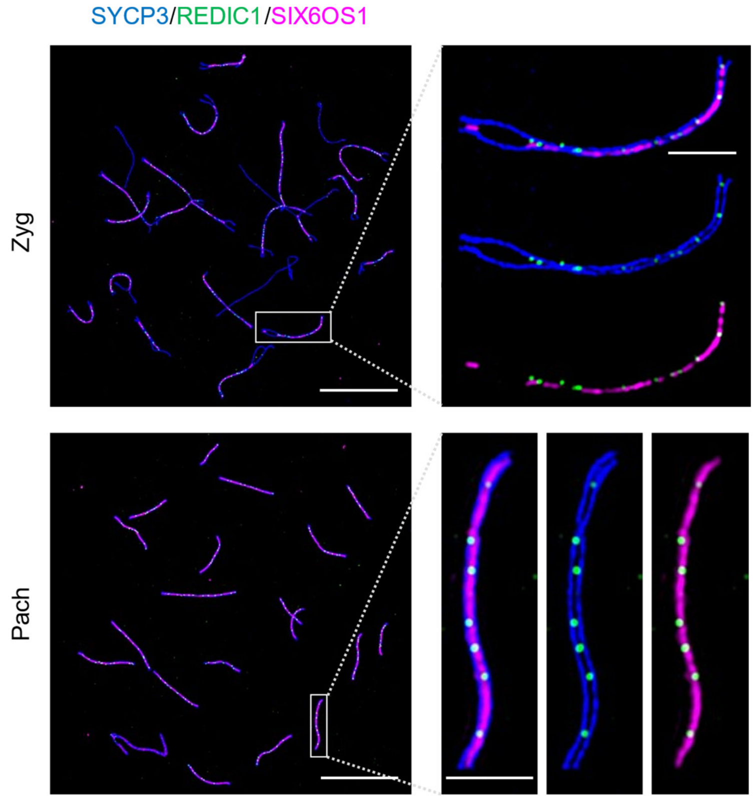

Supplementary Fig. S5 Structured illumination microscopy (SIM) of REDIC1 foci in spread mouse spermatocytes. The spread mouse spermatocytes from 30 dpp wild-type mice were stained for REDIC1 (green), SIX6OS1 (magenta), and chromosomal axis marker SYCP3 (blue). The areas in the white rectangles are enlarged on the right. Zyg, zygotene; Pach, pachytene. Images are representative of experiments from two male mice. Scale bars, 10 μm for the original images and 2 μm for the enlarged images, respectively.

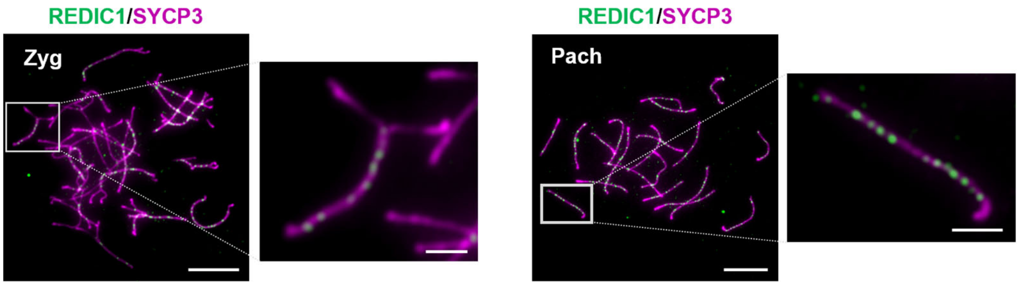

Supplementary Fig. S6 REDIC1 localizes to the paired regions of homologous chromosomes in zygotene and pachytene oocytes. Oocyte spreads were prepared from 18.5 dpc wild-type female mice and were immunostained for SYCP3 (magenta) and REDIC1 (green). The areas in the rectangles are magnified. Zyg, zygotene; Pach, pachytene. Images are representative of experiments from three wild-type female mice. Scale bars, 10 μm for the original images and 2 μm for the enlarged images, respectively.

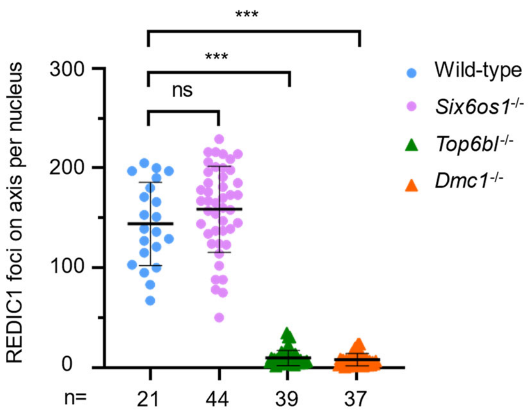

Supplementary Fig. S7 Quantification of REDIC1 foci on chromosome axes in spread zygotene spermatocytes of wild-type, *Six6os1*$^{-/-}$, *Top6bl*$^{-/-}$, and *Dmc1*$^{-/-}$ mice. n, the total number of nuclei analyzed. The bars indicate mean ± SD. P values were calculated by the Mann–Whitney test. ns, not significant; ****P* < 0.001.

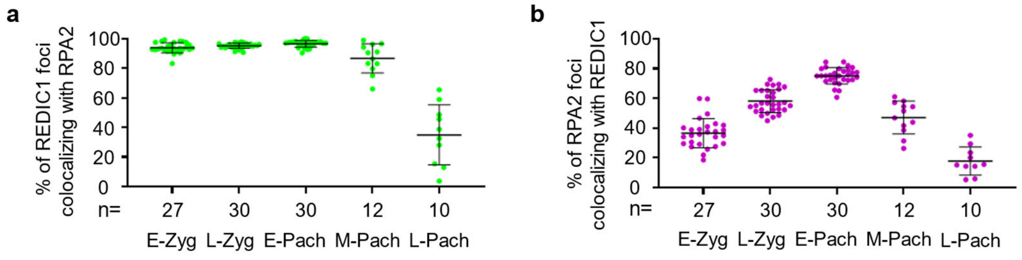

Supplementary Fig. S8 Quantification of REDIC1 and RPA2 colocalization. a Quantification of the percentage of REDIC1 foci colocalizing with RPA2 at indicated substages. **b** Quantification of the percentage of RPA2 foci colocalizing with REDIC1 at indicated substages. n, the total number of cells analyzed. Bars represent mean ± SD. E-Zyg, early zygotene; L-Zyg, late zygotene; E-Pach, early pachytene; M-Pach, mid-pachytene; L-Pach, late pachytene.

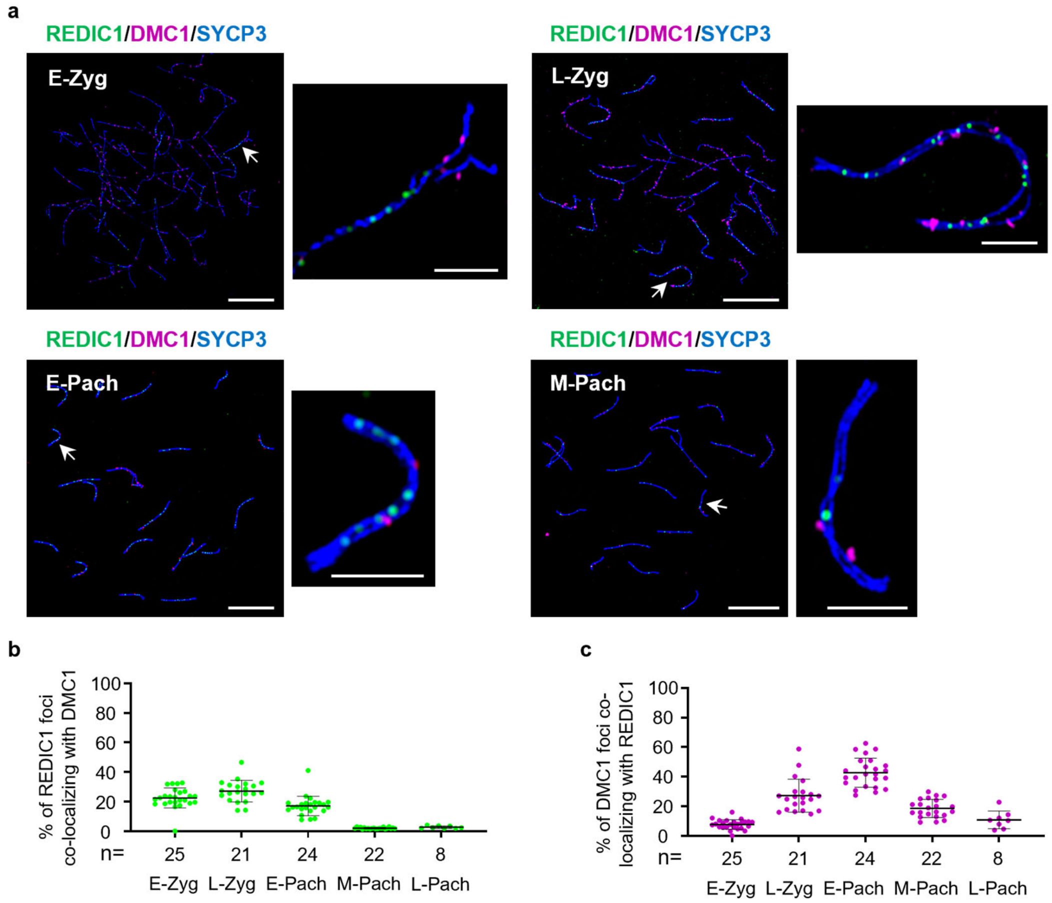

Supplementary Fig. S9 Colocalization analysis of REDIC1 and DMC1. a Representative images of structured illumination microscopy of spread zygotene and pachytene mouse spermatocytes stained for DMC1 (magenta), REDIC1 (green), and chromosomal axis marker SYCP3 (blue). The chromosomes indicated by the white arrow are magnified. Images are representative of experiments from two wild-type male mice. Scale bars, 10 μm for the original images and 2 μm for the enlarged images, respectively. **b** Quantification of the percentage of REDIC1 foci colocalizing with DMC1 at indicated substages. **c** Quantification of the percentage of DMC1 foci colocalizing with REDIC1 at indicated substages. n, the total number of cells analyzed. Bars represent mean ± SD. E-Zyg, early zygotene; L-Zyg, late zygotene; E-Pach, early pachytene; M-Pach, mid-pachytene; L-Pach, late pachytene.

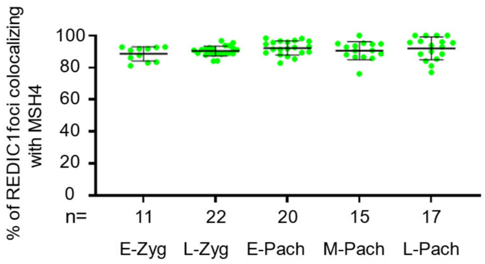

Supplementary Fig. S10 Quantification of REDIC1 and MSH4 colocalization at indicated substages. n, the total number of cells analyzed. Bars represent mean ± SD. E-Zyg, early zygotene; L-Zyg, late zygotene; E-Pach, early pachytene; M-Pach, mid-pachytene; L-Pach, late pachytene.

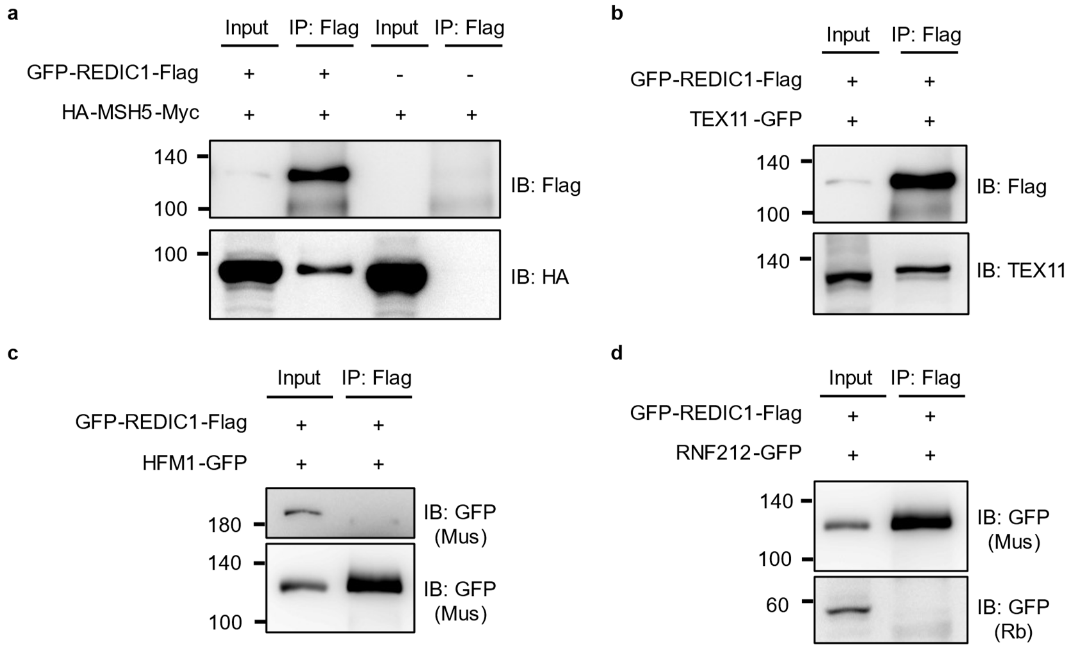

Supplementary Fig. S11 REDIC1 interacts with ZMM proteins MSH5 and TEX11. **a** Co-immunoprecipitation (co-IP) detecting the interaction between REDIC1 (fused with an N-terminal GFP tag and a C-terminal Flag tag) and MSH5 (fused with an N-terminal HA tag and a C-terminal Myc tag). **b** Co-IP detecting the interaction between REDIC1 and TEX11 (fused with a C-terminal GFP tag). **c** Co-IP detecting the interaction between REDIC1 and HFM1 (fused with a C-terminal GFP tag). **d** Co-IP detecting the interaction between REDIC1 and RNF212 (fused with a C-terminal GFP tag). HEK293T cells were transfected or co-transfected with the indicated expression vectors. Protein complexes were IPed with agarose beads coupled with anti-Flag antibodies and were analyzed by immunoblotting (IB) with the indicated antibodies. Mus, mouse-derived antibody; Rb, rabbit-derived antibody. The experiments were repeated three times. The primers used for the construction of the plasmids were listed in Supplementary Table. S6.

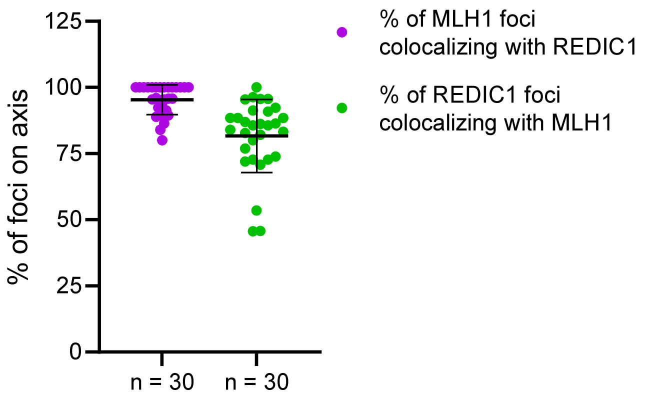

Supplementary Fig. S12 Quantification of REDIC1 and MLH1 colocalization in mid-pachytene spermatocytes. n, the total number of cells analyzed. Bars represent mean ± SD.

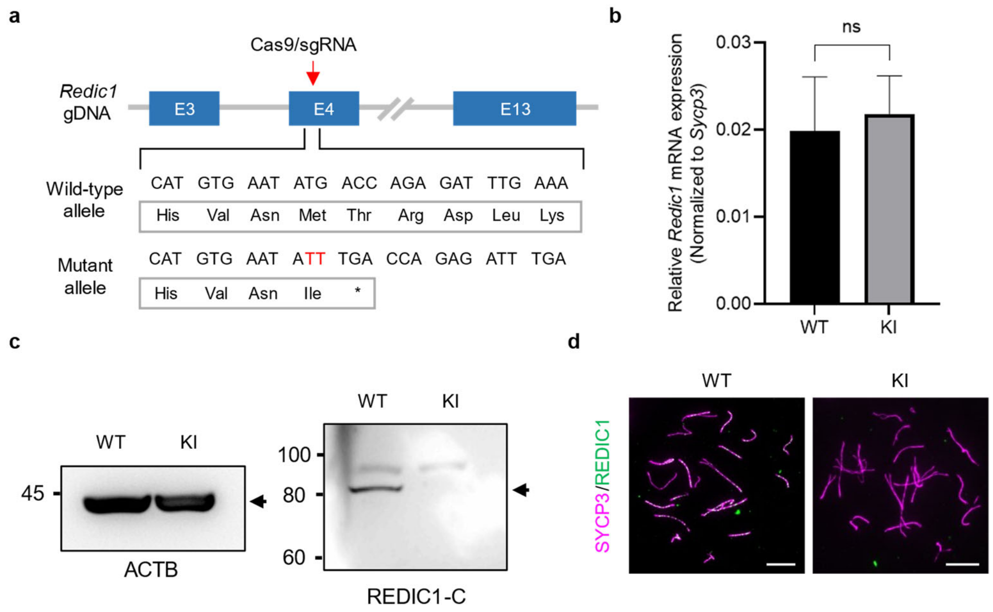

Supplementary Fig. S13 Generation of mouse models mimicking the patients' mutation by CRISPR/Cas9 technology. a A diagram of the genome editing strategy at the *Redic1* locus. The blue solid squares indicate exons. The expanded view of exon 4 shows the genomic DNA (gDNA) and corresponding protein sequences of the edited region in wild-type and mutant alleles. The red-colored characters indicate the 2-bp insertion and the asterisk shows the premature stop codon in *Redic1* KI mice. **b** RT–qPCR analysis of *Redic1* expression at the cDNA level in testis tissues from wild-type (WT) and *Redic1* KI mice. *Sycp3* served as the reference gene. The experiments were performed in triplicate and the data represent the mean ± SD. ns, not significant; two-tailed unpaired *t*-test. **c** Western blot of testicular lysates from 20 dpp wild-type and homozygous *Redic1* KI mice using REDIC1 antibody. ACTB served as the internal control. The arrows indicate the target bands. **d** Immunostaining of spermatocyte spreads from WT and *Redic1* KI mice with SYCP3 (magenta) and REDIC1 (green) antibodies. Scale bars, 10 μm.

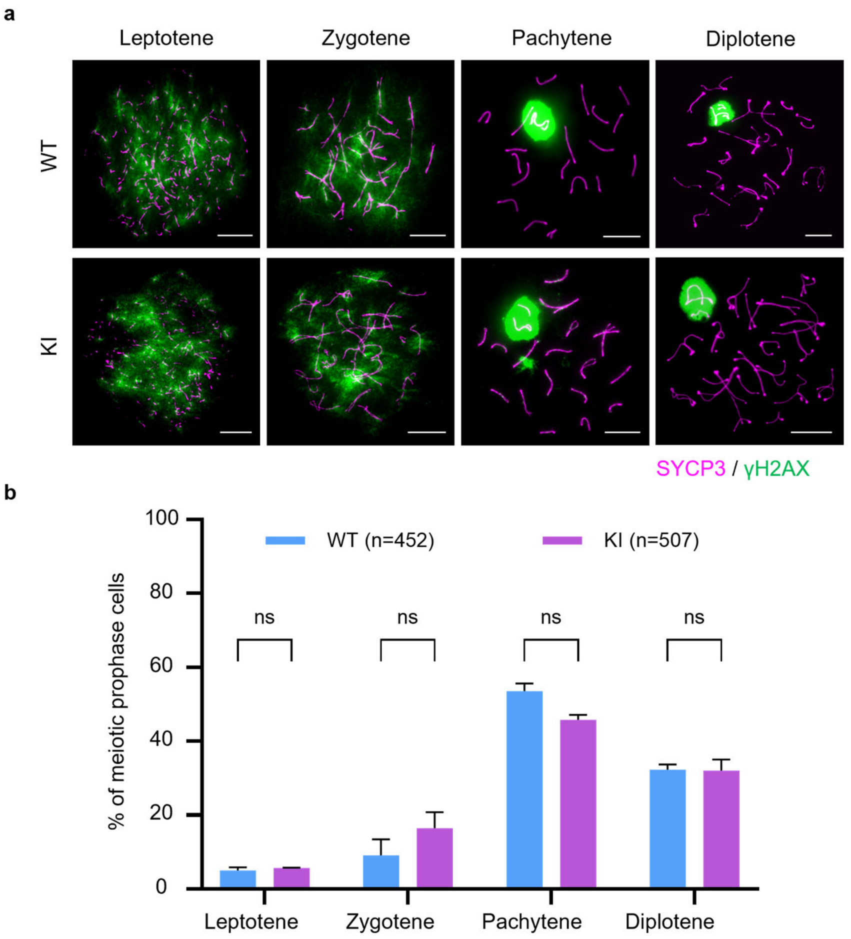

Supplementary Fig. S14 Meiotic prophase I analysis. a Representative images of spread spermatocytes from 2-month-old wild-type (WT) and *Redic1* KI mice were stained with antibodies against SYCP3 (magenta) and γH2AX (green). Scale bars, 10 µm. **b** The proportions of spermatocytes at the indicated substages. The data are from two independent experiments and the bars represent the mean ± SD. The "n" indicates the number of meiotic prophase cells examined. ns, not significant ($P > 0.05$), two-way ANOVA. *Redic1* KI mice represent mice carrying the homozygous mutation c.232_233insTT in *Redic1*.

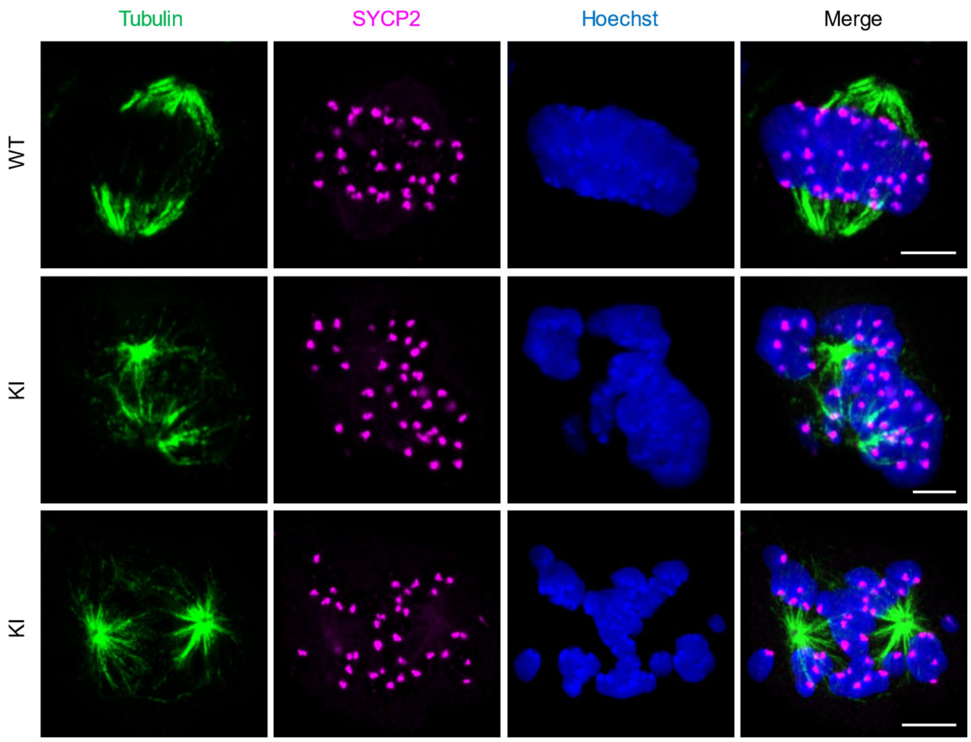

Supplementary Fig. S15 Analyses of the spindle morphology of the metaphase I spermatocytes. Spermatocyte smears were prepared from ~20 dpp wild-type (WT) and adult *Redic1* KI mice, and immunostained with Tubulin (green) and SYCP2 (magenta) antibodies. The nuclei were stained with Hoechst 33342 (blue). The experiments were repeated two times with similar results. Scale bars, 10 μm.

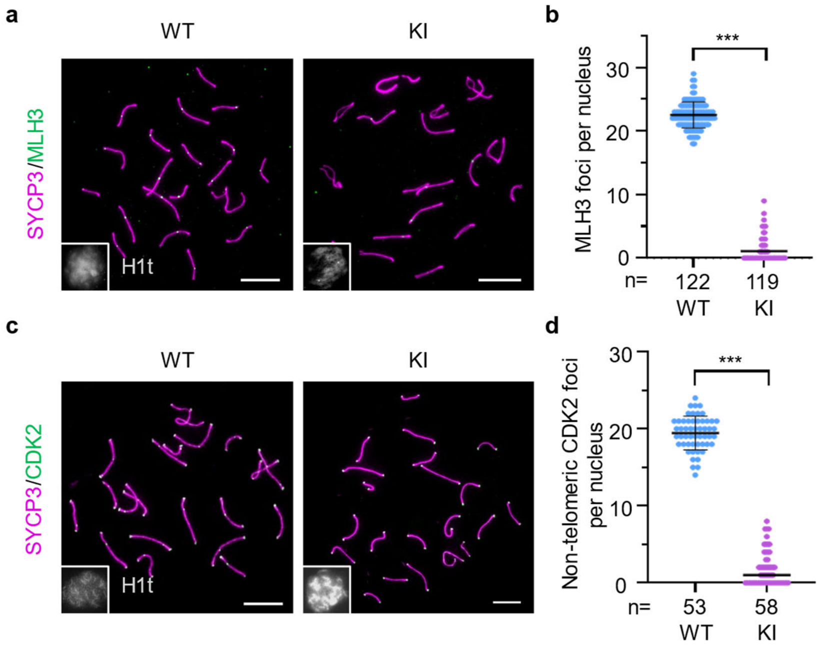

Supplementary Fig. S16 Crossover markers MLH3 and CDK2 are largely reduced in *Redic1* KI mice. a Representative spreads of mid-pachytene spermatocytes from WT and *Redic1* KI mice stained for SYCP3 (magenta), MLH3 (green), and H1t (white in the insets). Scale bars, 10 µm. **b** Quantification of MLH3 foci on the axis in mid-pachytene spermatocytes. n, the total number of cells analyzed from three animals for each genotype. Bars represent the mean ± SD. ***$P < 0.001$; Mann–Whitney test. **c** Representative spreads of mid-pachytene spermatocytes from WT and *Redic1* KI mice stained for SYCP3 (magenta), CDK2 (green), and H1t (white in the insets). Scale bars, 10 µm. **d** Quantification of non-telomeric CDK2 foci in mid-pachytene spermatocytes. n, the total number of cells analyzed from two animals for each genotype. Bars represent the mean ± SD. ***$P < 0.001$; Mann–Whitney test. *Redic1* KI mice represent mice carrying the homozygous mutation c.232_233insTT in *Redic1*.

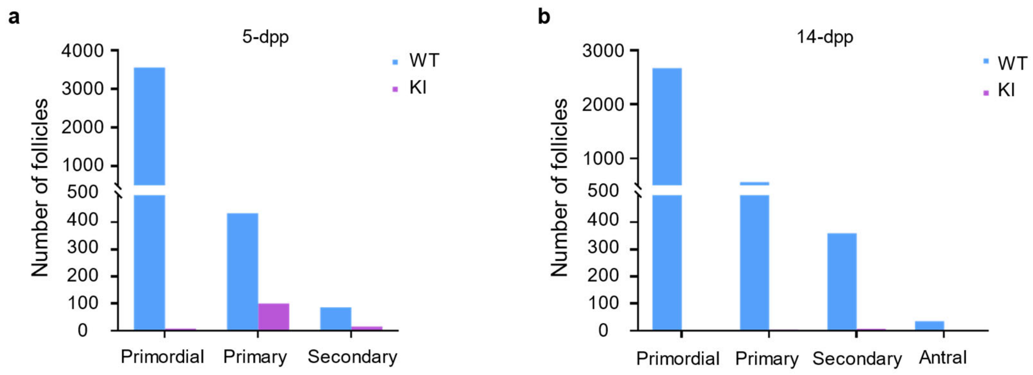

Supplementary Fig. S17 Quantification of the number of follicles in the ovary of wild-type and *Redic1* KI mice at indicated ages. a The number of follicles in the ovary of 5-dpp WT and *Redic1* KI female mice. **b** The number of follicles in the ovary of 14-dpp WT and *Redic1* KI female mice. The data are from one animal for each genotype.

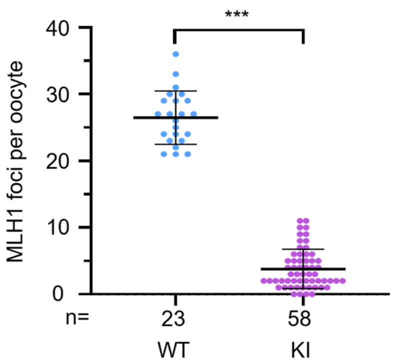

Supplementary Fig. S18 Quantification of MLH1 foci in oocytes from WT and *Redic1* KI female mice. n, the total number of cells analyzed. Bars represent the mean ± SD. P value was calculated by the Mann–Whitney test. ***$P < 0.001$.

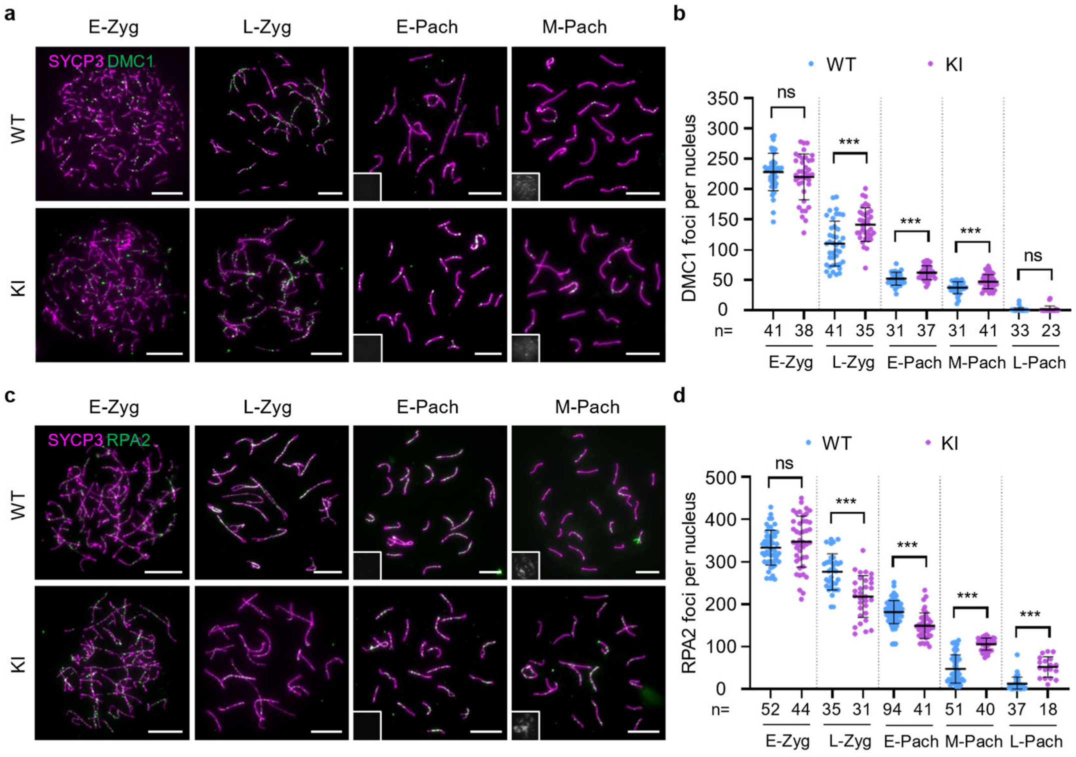

Supplementary Fig. S19 DSB repair is delayed in *Redic1* mutant cells. a Representative spread spermatocytes from wild-type (WT) and *Redic1* KI mice were immunostained for SYCP3 (magenta) and DMC1 (green). Early zygotene, late zygotene, early pachytene, and mid-pachytene spermatocytes are shown. Scale bars, 10 μm. **b** Quantification of DMC1 foci in spread spermatocytes at the indicated substages. **c** Representative spread spermatocytes from WT and *Redic1* KI mice were immunostained for SYCP3 (magenta) and RPA2 (green). Early zygotene, late zygotene, early pachytene, and mid-pachytene spermatocytes are shown. Scale bars, 10 μm. **d** Quantification of RPA2 foci in spread spermatocytes at the indicated substages. H1t staining was used to differentiate the substages of pachytene and shown in the insets of **a** and **c**. The "n" in **b** and **d** shows the number of nuclei analyzed from at least two animals. The bars indicate the mean ± SD. P values were calculated by the Mann–Whitney test. ns, not significant ($P > 0.05$); ***$P < 0.001$. E-Zyg, early zygotene; L-Zyg, late zygotene; E-Pach, early pachytene; M-Pach, mid-pachytene; L-Pach, late pachytene. *Redic1* KI mice represent mice carrying the homozygous mutation c.232_233insTT in *Redic1*.

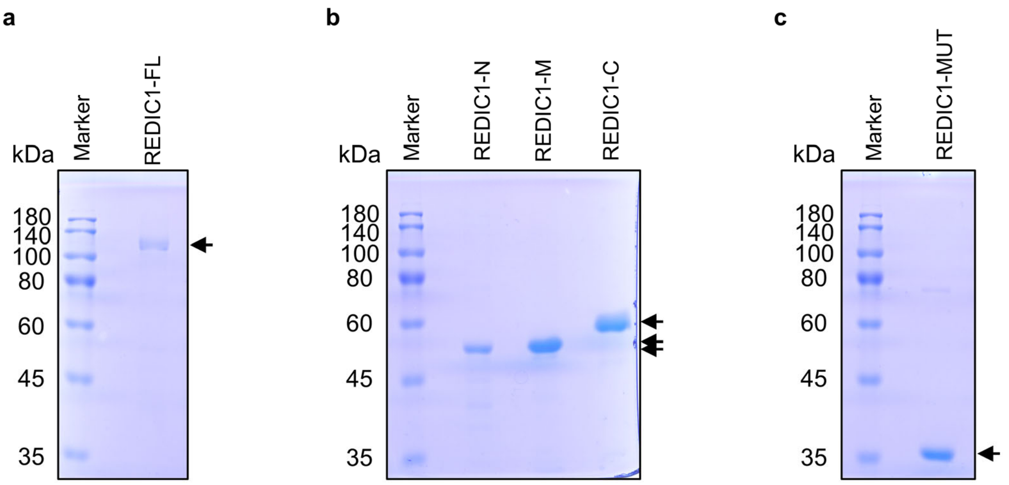

Supplementary Fig. S20 SDS–PAGE of purified REDIC1 proteins. The proteins have N-terminal GST and C-terminal Flag tags. They were expressed in *Escherichia coli* cells and subjected to GST and Flag affinity purification. The 9% polyacrylamide gel was stained with Coomassie brilliant blue. The bands for target proteins are indicated by arrows.

Supplementary Table S1. Clinical characteristics of the affected individuals

	Reference values	P2273	P7452
Age (year)[a]	–	40	30
Height/Weight (cm/kg)	–	175/75	180/95
Karyotype	–	46, XY	46, XY
Diagnosis of disease	–	NOA	NOA
**Physical examination**[b]			
Testis size (mL)	>12.5	5	–
**Semen analysis**[c]			
Semen volume (mL)	⩾1.4	2	–
Sperm concentration (10^6/mL)	⩾16	0	0
**Hormone analysis**[d]			
FSH (U/L)	0.80—5.10	2.19	–
LH (U/L)	1.24—8.62	7.57	–
Prolactin (ng/mL)	2.64—13.13	6.28	–

NOA, nonobstructive azoospermia; FSH, follicle-stimulating hormone; LH, luteinizing hormone.

[a]Ages at the manuscript submission.

[b]Physical examination was performed by a consultant gynecologist.

[c]Reference values were published by WHO in 2021.

[d]Reference values were suggested by the local hospital.

Supplementary Table S2. Primers used for the generation of mutant mouse models

Primer name	Sequence (5'-3')	Product (bp)
Redic1-KI-sgRNA-Fw	GAAATTAATACGACTCACTATAGGGAGATAGAGGGACTTTCAAATCTCGTTTTAGAGC	122
Redic1-KI-sgRNA-Rv	AAAAAAGCACCGACTCGGTG	
Redic1-KI-oligo	GTAAATCAGATCTCGTCCATGAAGGAAAATTCTGAAACCATGAAAAGACCAACTCATGTGAATATTTGACCAGAGATTTGAAAGTCCCTCTAAGGAAGCATGATTTAGAACTTCCAATGTCACCTCACTG	–
Redic1-check-Fw	ATGAAAGGGTCGTCTGACCT	634 (WT)<br>636 (KI)
Redic1-check-Rv	GGGTGTGCATCCTAACAGTT	
Six6os1-KO-sgRNA-Fw	GAAATTAATACGACTCACTATAGGGAGAAGCTATAAACAAAGTAGATGGTTTTAGAGC	122
Six6os1-KO-sgRNA-Rv	AAAAAAGCACCGACTCGGTG	
Six6os1-exon4-Fw	AGGTTCTTACTTTATGTATGCTCT	544 (WT)<br>539 (KO)
Six6os1-exon4-Rv	TACAAGTTGCCCTAAGCAGT	
Redic1-Flag/Myc-sgRNA-Fw	GAAATTAATACGACTCACTATAGGGAGATAGAGGGACTTTCAAATCTCGTTTTAGAGC	122
Redic1-Flag/Myc-sgRNA-Rv	TGTTAGAGTGAGTTACTTGT	
Redic1-Flag/Myc-donor (Only insertion sequence shown)	GACTACAAAGACCATGACGGTGATTATAAAGATCATGATATCGATTACAAGGATGACGATGACAAGGGATCCGAACAAAAACTCATCTCAGAAGAGGATCTGGAACAAAAACTCATCTCAGAAGAGGATCTGGAACAAAAACTCATCTCAGAAGAGGATCTG	–
Redic1-Flag/Myc-Fw	CTTGTGGGGAACACAGCAGA	294 (WT)<br>456 (KI)
Redic1-Flag/Myc-Rv	CCCCCTTTATGTTCAGCCTCA	

Supplementary Table S3. The antibodies used in this study

Primary antibodies

Target	Dilution	Host	Supplier	Catalog number
Human SYCP3	1:200	Mouse	Proteintech	66409-1-Ig
Human C14ORF39-C	1:30	Guinea pig	Made by Abclonal	–
Human MLH1	1:200	Mouse	BD biosciences	550838
Mouse SYCP3	1:200	Mouse	Abcam	ab97672
Mouse SYCP1	1:200	Rabbit	Novus Biologicals	NB300-229
Mouse SIX6OS1	1:300	Rat	Made by Abclonal	–
Mouse MLH1	1:100	Rabbit	Made by Abclonal	–
Mouse MLH3	1:300	Rabbit	Gifted by Mengcheng Luo's lab	–
Mouse CDK2	1:50	Mouse	Santa Cruz	sc-6248
Mouse MSH4	1:50	Rabbit	Made by Duoneng	–
Mouse TEX11	1:50	Rabbit	Gifted by P. Jeremy Wang's lab	–
Mouse DMC1	1:50	Guinea pig	Made by Abclonal	–
Mouse RNF212	1:50	Rabbit	Gifted by Mengcheng Luo's lab	–
Mouse HEI10	1:300	Rabbit	Gifted by Hongbin Liu's lab	–
Mouse SYCP2	1:200	Guinea pig	Made by Abclonal	–
Mouse RPA2	1:100	Rat	Cell Signaling Technology	2208
Mouse REDIC1-C	1:100 (for IF)<br>1:500 (for WB)	Rabbit	Made by Dia-an	–
Mouse H1t	1:500	Guinea pig	Made by Dia-an	–
Tubulin	1:500	Rabbit	Abcam	ab6046
γH2AX	1:5000	Rabbit	Novus Biologicals	NB100-384
GFP	1:2000 (for WB)	Mouse	Abmart	M20004
GFP	1:3000 (for WB)	Rabbit	DUONENG-BIO	AB01020
HA-Tag Conjugated HRP	1:2000	Mouse	Cell Signaling Technology	2999S
Flag-Tag	1:100 (for IF)<br>1:2000 (for WB)	Mouse	Proteintech	66008-2-Ig
GAPDH	1:5000 (for WB)	Mouse	Proteintech	60004-1-Ig

Secondary antibodies

Target	Dilution	Host	Supplier	Catalog number
Goat anti-mouse IgG (Alexa-488)	1:100	Goat	Molecular Probes	A-21121
Goat anti-mouse IgG (Alexa-647)	1:100	Goat	Invitrogen	A-21235
Donkey anti-rabbit IgG (Alexa-488)	1:100	Donkey	Thermofisher	A-21206
Donkey anti-rabbit IgG (Alexa-555)	1:200	Donkey	Molecular Probes	A31572
Goat anti-rat IgG (Alexa-568)	1:200	Goat	Thermofisher	A11077
Goat anti-Guinea Pig IgG (Alexa-555)	1:150	Goat	Thermofisher	A21435
Goat anti-Guinea Pig IgG (Alexa-647)	1:100	Goat	Thermofisher	A21450
Goat anti-mouse IgG (HRP)	1:10000	Goat	Biolegend	405306
Donkey anti-rabbit IgG (HRP)	1:10000	Donkey	Biolegend	406401

Supplementary Table S4. Primers for the construction of REDIC1 expression plasmids

Primer name	Sequence (5'-3')
GST-REDIC1 FL-Flag-F	ACCTGTATTTTCAGGGATCGATGAACTGGGTCGGGGGCTC
GST-REDIC1 FL-Flag-R	TCATCGTCTTTGTAATCCGAGAGTGAGTTACTTGTAGGTGTTTCCTGCC
GST-REDIC1 N-Flag-F	CGCCATACAAGCGAACAAACTCGGATTACAAAGACGATGACG
GST-REDIC1 N-Flag-R	TCATCGTCTTTGTAATCCGAGTTTGTTCGCTTGTATGGCG
GST-REDIC1 M-Flag-F	CAAACAGTCATGTGATTTTGACTCGGATTACAAAGACGATGACG
GST-REDIC1 M-Flag-R	TCATCGTCTTTGTAATCCGAGTCAAAATCACATGACTGTTTG
GST-REDIC1 C-Flag-F	ACCTGTATTTTCAGGGATCGCAGAATGAGATCCCAATGGA
GST-REDIC1 C-Flag-R	TCCATTGGGATCTCATTCTGCGATCCCTGAAAATACAGGT
GST-REDIC1 Mut-Flag-F	GAAAAGACCAACTCATGTGAATAttTCGGATTACAAAGACGATGACG
GST-REDIC1 Mut-Flag-R	TCATCGTCTTTGTAATCCGAaaTATTCACATGAGTTGGTCTTTTC
GST-REDIC1-Flag AS-F	TGCGGTATTTTCTCCTTACG
GST-REDIC1-Flag AS-R	CGTAAGGAGAAAATACCGCA
GST-Flag-Vector-F	TCGGATTACAAAGACGATGACG
GST-Flag-Vector-R	CGATCCCTGAAAATACAGGTTTTCATC

Supplementary Table S5. Oligonucleotides used in electrophoretic mobility shift assays

Primer name	Sequence (5’-3’)	
PC1253 (5’-FAM)	TGGGTCAACGTGGGCAAAGATGTCCTAGCAATGTAATCGTCTATGACGTT	ssDNA
PC1253 (5’-FAM)	TGGGTCAACGTGGGCAAAGATGTCCTAGCAATGTAATCGTCTATGACGTT	Holliday Junction
PC1254	TGCCGAATTCTACCAGTGCCAGTGATGGACATCTTTGCCCACGTTGACCC	
PC1255	GTCGGATCCTCTAGACAGCTCCATGATCACTGGCACTGGTAGAATTCGGC	
PC1256	CAACGTCATAGACGATTACATTGCTACATGGAGCTGTCTAGAGGATCCGA	
PC1253 (5’-FAM)	TGGGTCAACGTGGGCAAAGATGTCCTAGCAATGTAATCGTCTATGACGTT	dsDNA
PC1253C	AACGTCATAGACGATTACATTGCTAGGACATCTTTGCCCACGTTGACCCA	
BB (5’-FAM)	TCAAGCTCGGTCTGCAGTCAGGATGATTGTGAGCGTTAACCCTAACCCTAA CCCTAACCCTAATCTGCACTCGAGACTCACGTCCTGGTCACG	D-loop
BT	CGTGACCAGGACGTGAGTCTCGAGTGCAGACCTTTTTTTTTTTTTTTTTTTT TTTTTTTTTTTACAATCATCCTGACTGCAGACCGAGCTTG	
INVa	CACCATCCAGTTCTCTTCGCGGC	
INVb	GCCGCGAAGAGAACTGGATGGTGTTAGGGTTAGGGTTAGGGTTAGGGTTA ACGCTC	

Note: These oligo sequences are from the reported paper[5].

Supplementary Table S6. Primers used for the construction of plasmids in Co-IP assays

Primer name	Sequence (5'-3')	Product (bp)
GFP-Gene-Flag-vec-Fw	AACTCTGCTCCGAGAGCCCCCGACCCAGTTCTTGTACAGCTCGTCCATGC	4787
GFP-Gene-Flag-vec-Rv	GATTACAAAGACGATGACGATAAATAAAGCGGCCGCGACTCTAGATCATA	
GFP-*Redic1*-Flag-Fw	GCATGGACGAGCTGTACAAGAACTGGGTCGGGGGCTCTCG	2000
GFP-*Redic1*-Flag-Rv	TTATTTATCGTCATCGTCTTTGTAATCGAGTGAGTTACTTGTAGGTGTTTCCTGC	
HA-Gene-Myc-vec-Fw	GAGCAGAAGCTGATCTCAGAGGAGGACCTGTAAAGCGGCCGCGACTCTAGATC	3994
HA-Gene-Myc-vec-Rv	AGCGTAATCTGGTACGTCGTATGGGTACATGCTAGCGGATCTGACGGTTC	
HA-*mMsh5*-Myc-Fw	ATGTACCCATACGACGTACCAGATTACGCTGCTTTCAGAGCGACCCCAGG	2556
HA-*mMsh5*-Myc-Rv	CAGGTCCTCCTCTGAGATCAGCTTCTGCTCGAGGATGGTGGGAGCAGCGG	
GFP-linker-Gene-vec-Fw	AGCGGCCGCGACTCTAGATC	4751
GFP-linker-Gene-vec-Rv	TCCTGCAGCTCCACCGCTCGACTTGTACAGCTCGTCCATGC	
GFP-linker-*mTex11*-Fw	TCGAGCGGTGGAGCTGCAGGAGACCGCATTACTGACTTTTACTT	2882
GFP-linker-*mTex11*-Rv	GATCTAGAGTCGCGGCCGCTTTACAGATGGTTTTGAGCTGC	
GFP-Gene-vec-Fw	ATGGTGAGCAAGGGCGAGGA	4651
GFP-Gene-vec-Rv	GCTAGCGGATCTGACGGTTC	
GFP-*mRnf212*-Fw	GAACCGTCAGATCCGCTAGCATGGCCAGCTGGGTGTTCTG	961
GFP-*mRnf212*-Rv	TCCTCGCCCTTGCTCACCATCCGATGAACGCATGCATGCC	
GFP-*mHfm1*-Fw	GAACCGTCAGATCCGCTAGCATGCCAAAGTCAGACGATTG	4342
GFP-*mHfm1*-Rv	TCCTCGCCCTTGCTCACCATGAAAATGCCATTAAATATTCCCAGAAG	

Supplementary Methods

Real-time quantitative polymerase chain reaction (RT–qPCR)

Total RNAs were extracted from fresh mouse tissues using RNAiso Plus reagents (TaKaRa, 9109) and cDNAs were synthesized with the PrimeScript RT reagent kit (TaKaRa, RR047A) following the manufacturer's protocols. RT–qPCR was performed with Hieff® qPCR SYBR Green Master Mix (Yeasen, 11201ES03) and a LightCycler® 96 qPCR instrument (Roche). The following primers were used: *Redic1*, 5'-TCTCGGAGCAGAGTTCTGAT-3' (forward) and 5'-TGTGATGATTGAACTGG-GCC-3' (reverse); *Actb*, 5'-CATTGCTGACAGGATGCAGAAGG-3' (forward) and 5'-TGCTGGAAGGTGGACAGTGAGG-3' (reverse); and *Sycp3*, 5'-GGACTGTA-TTTACTCCTGCCCAA-3' (forward) and 5'-TTCTTCCACCAGGCACCATCT-3'.

Cell transfections

HEK293T cells were cultured in plastic dishes in DMEM supplemented with 10% fetal bovine serum and 1% penicillin/streptomycin at 37°C in a 5% CO_2 incubator. Cells were plated 12 hours in advance, and plasmid transfection was performed using Lipofectamine 3000 (Thermo Fisher, L300015) at ~70% confluency according to the manufacturer's instructions. After 36 hours of transfection, cells were harvested and directly lysed with 2× SDS sample buffer for Western blot analysis.

Co-immunoprecipitation (co-IP)

HEK293T cells were transfected or co-transfected with the indicated expression vectors. After 48 hours of transfection, cells were harvested and lysed with 500 µl IP buffer (50 mM Tris pH 7.4, 150 mM NaCl, 0.5% Triton X-100, 3 mM $MgCl_2$. 10% Glycerol, 1 mM DTT, and Protease Inhibitor Cocktail). Take 50 µl of lysate as input sample, add 35 µl of agarose beads conjugated with Flag antibody (Genscript, L00432) to the remaining lysate, and incubate at 4°C with slow rotation for 6 hours. After 4 washes with IP buffer, the protein complexes were directly lysed with 2× SDS sample buffer for Western blot analysis.

Spermatocyte smears preparations and immunofluorescence

Testes from ~20 dpp wild-type and adult *Redic1* KI mice were dissected and separated from tunica. The testicular cell suspension was made by a mechanical method. PBS was added to the cell suspension to bring the volume to 5 ml and then centrifuged at 100 g for 5 min at room temperature. Discard the supernatant and gently resuspend the cell pellet with 200 µl of fetal bovine serum. Add 20 µl of the cell suspension to one side of

the glass slide and use a coverslip to spread the cell suspension gently to the other side. After air-drying, the smear slides are ready for immunostaining.

The slides were fixed in 4% PFA for 10 min. The slides were fixed in 4% paraformaldehyde for 10 min and then permeabilized with 0.5% PBST (Triton X-100) for 5 min. After being blocked with 3% BSA for 30 min, the primary antibody was applied and incubated overnight at 4°C. The slides were washed 3 times in 0.03% PBST and the secondary antibodies and Hoechst 33342 were applied and incubated at 37°C for 1 hour. After 3 washes, the slides are applied with the appropriate amount of VECTASHILED and finally covered with a clean coverslip. The images were obtained using a Nikon C2plus laser confocal microscope with five layers captured in a Z-axis step of 0.5 μm and then projected at maximum signal intensity using the NIS-Elements software (Nikon).

Preparation of testicular protein extracts and Western blot

Testicular tissues were homogenized in appropriate amounts of lysis buffer (50 mM Tris-HCl (pH 7.5), 150 mM NaCl, 0.5% Triton X-100, 1 mM EDTA, 10% glycerol, protease inhibitor cocktail) on ice using the tissue grinding tubes. The homogenate was transferred to a 1.5 ml Eppendorf tube for sonication, followed by centrifugation at 16,000 g for 10 min at 4°C. The supernatants were mixed with 1/5 volume 5× SDS sample buffer and boiled for 10 min at 100°C. Protein samples were separated by 9% SDS–PAGE and then blotted onto nitrocellulose membranes, blocked with 5% skim milk, and then primary antibody was applied overnight at 4°C. After three washes in TBST (0.1% Triton X-100), HRP-coupled secondary antibodies were applied for 1 hour at room temperature. After three washes, the signals were obtained on the ImageQuant LAS4000 instrument (GE Healthcare) using the enhanced chemiluminescence method. The antibodies used are listed in Supplementary Table. S3.

Supplementary References

1. Notredame, C., Higgins, D.G. & Heringa, J. T-Coffee: A novel method for fast and accurate multiple sequence alignment. *J Mol Biol* **302**, 205-17 (2000).
2. Robert, X. & Gouet, P. Deciphering key features in protein structures with the new ENDscript server. *Nucleic Acids Res* **42**, W320-4 (2014).
3. Combet, C., Blanchet, C., Geourjon, C. & Deleage, G. NPS@: network protein sequence analysis. *Trends Biochem Sci* **25**, 147-50 (2000).
4. Letunic, I., Khedkar, S. & Bork, P. SMART: recent updates, new developments and status in 2020. *Nucleic Acids Res* **49**, D458-D460 (2021).
5. Cannavo, E. *et al.* Regulation of the MLH1-MLH3 endonuclease in meiosis. *Nature* **586**, 618-622 (2020).
